# Hydrothermal synthesis, characterization and enhanced photocatalytic activity and toxicity studies of a rhombohedral Fe_2_O_3_ nanomaterial†

**DOI:** 10.1039/c9ra04978a

**Published:** 2019-08-13

**Authors:** Mavinakere Ramesh Abhilash, Akshatha Gangadhar, Jagadish Krishnegowda, Mahendra Chikkamadaiah, Shivanna Srikantaswamy

**Affiliations:** Department of Studies in Environmental Science, University of Mysore Manasagangotri Mysore 570006 India srikantas@hotmail.com abhilash@envsci.uni-mysore.ac.in; Centre for Materials Science and Technology, Vijnana Bhavan, University of Mysore Manasagangotri Mysore 570006 India; Department of Studies in Botany, University of Mysore Manasagangotri Mysore 570006 India

## Abstract

The present investigation focuses on the synthesis of metal oxide nanoparticles (MONPs) *via* a facile hydrothermal route. The material has been characterized by using X-ray diffractometry (XRD), X-ray fluorescence (XRF), Fourier-transform infrared spectroscopy (FTIR), dynamic light scattering (DLS), high resolution transmission electron microscopy (HR-TEM), energy dispersive spectroscopy (EDS), photoluminescence (PL), atomic force microscopy (AFM) and Brunauer–Emmett–Teller (BET) techniques. However, the application of Fe_2_O_3_ metal oxide nanoparticles (MONPs) tied with their inimitable chemical and physical nature is thought to emphasize their exploitable medical and biological applications nowadays. Rhodamine-B (RB) was used for photocatalytic degradation studies by using rhombohedral Fe_2_O_3_, afterwards the material was recycled and utilized for toxicity assessments. Undeniably, a meticulous assessment is needed of the factors that influence the biocompatibility and is essential for the safe and sustainable development of the emerging chemically synthesized metal oxide nanoparticle (MONPs). The toxicity assessment of Fe_2_O_3_ is necessary to know the bioaccumulation and local or systemic toxicity associated to them. The aim of the present study is to investigate the effects of Fe_2_O_3_ and its histological alterations of the heart tissue of albino Wistar rat. The synthesized materials high dose was found to be highly stable and we found more toxicity against the skin melanoma cells (B16-F10), human embryonic kidney (HEK), 293 cells depending on dose. Finally, *Escherichia coli*, (MTCC 7410) bacterial cell wall damage studies were also conducted to provide a clear determination of rhombohedral nanomaterial behaviour. The fusion of these biocompatibility investigations paves a way for further applications in utilization of these materials in future eco-friendly applications.

## Abstract 2

The present investigation focuses on the synthesis of metal oxide nanoparticles (MONPs) *via* a facile hydrothermal route.

## Graphical abstract

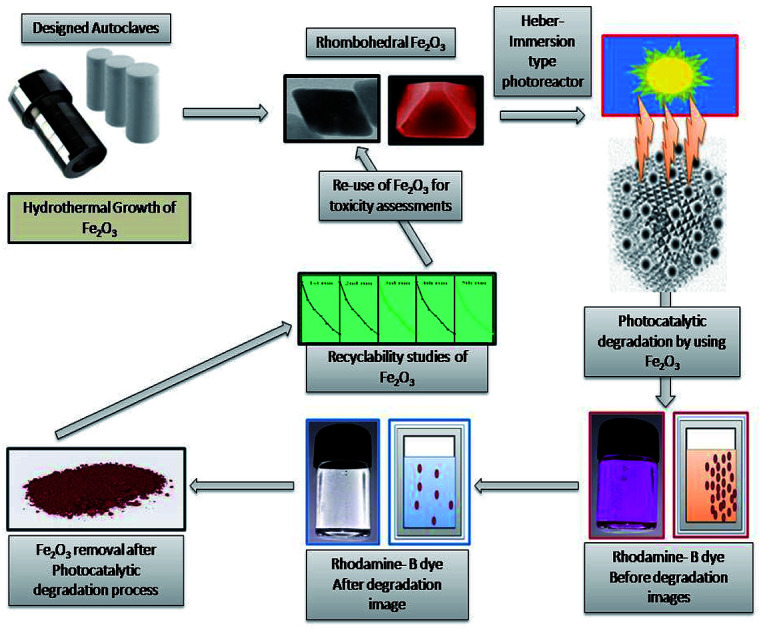

## 1 Introduction

**Fig. 1:**
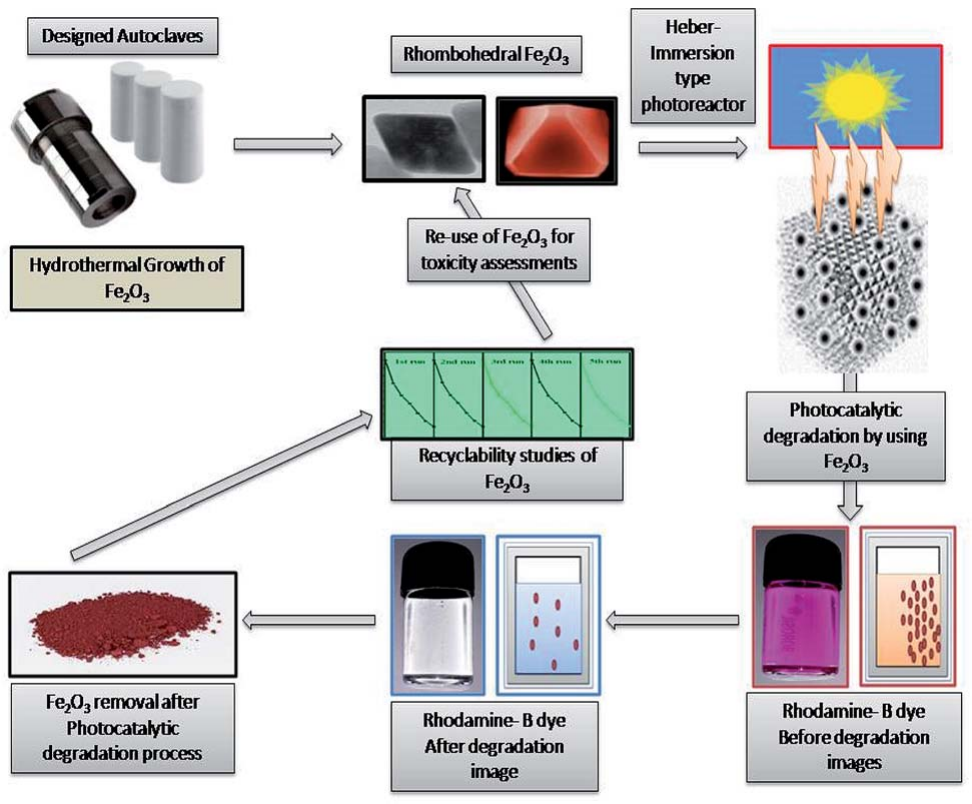
Hydrothermal growth of rhombohedral Fe_2_O_3_ degradation and recyclability studies.

There is a growing interest about the strong associations between exposure to nanoparticles and adverse health effects in humans.^1^ For this strong reason, environmental contamination by nanoparticles is attracting considerable and increasing global concern.^2–5^ The behaviour and toxicity of particles mainly comes from studies on orally administrated MONPs.^6,7^ (MONPs) may differ in reactivity and solubility and may interact with all kinds of endogenous proteins, lipids, polysaccharides and cells and series of tests were anticipated for systematic evaluation of the toxicity of (MONPs) used in drug delivery systems.^8,9^ Medical applications of Fe_2_O_3_ metal oxide nanoparticle (MONPs) are wider than others due to biocompatibility nature, high stability and ease of use and have a more applications in drug delivery.^10,11^ Metal oxide photocatalysis is an enthralling application tool for solar energy conversion and dye degradation due to its prominent nature.^12^ Because of widespread application of these particles in various industries human exposition to them increased, so investigation of this nanoparticle role in cell growth and survival has more importance.^13^ Iron oxide is the most stable under ambient conditions to perform various applications with environment friendly label with a band gap of 2.1 eV with tuneable nature,^14–16^ there are many methods used for the synthesis of Fe_2_O_3_ metal oxide nanoparticle (MONPs) like sol–gel method,^17^ hydrothermal and solvothermal synthesis,^14^ thermal breakdown,^18^ and sonochemical synthesis,^19^ have been used for synthesis of Fe_2_O_3_ with different morphologies and concerned nanostructures. Human skin, heart, lungs and digestive system are the most common entry routes for nanoparticles and its pathogenicity.^20^ The airborne nanoparticles have high mobility and can be inhaled into the respiratory system easily.^21^ One of the most common damaging effects of nanoparticles are rise of reactive oxygen species (ROS) that refer to oxidative stress in human and animal tissues. It's confirmed that almost all of the studied nanoparticle produce reactive oxygen species and it is the main mechanism for nanoparticle toxicity that can lead to inflammation and apoptosis.^22^ The unique properties and interactions between nanomaterials with biological system are essential.^23,24^ Cell line toxicity of human cell lines by using several synthesized metal oxides are more attractive in understanding the relationship between nanoparticle–cell interaction as well as in biocompatibility studies.^25,26^ Furthermore, biocompatibility must be conducted with particular focus on the environment in which the nanomaterial will be placed in.^27^ Size, shape, and morphology of nanoparticles, play a vital role in mammalian cell culture medium and the nanoparticle are much more toxic than the hydrothermally synthesized nanocomposite semiconductor.^28^ The drug delivery, evaluation of metal oxide nanomaterial biocompatibility is essential to secure drug release with low cytotoxicity.^29,30^ The biocompatibility evaluation of structured nanoparticles for drug delivery applications has been expanded from being primarily investigated in a laboratory setting to being applied in the multi-billion-dollar pharmaceutical industry.^31–34^ The rhombohedral Fe_2_O_3_ (MONPs) are effectively utilized for degradation of rhodamine-B (RB-B) degradation process nanomaterial was successfully recycled and utilized for toxicity assessment potentials. After the degradation studies, it is a researcher duty to assess the toxicity of material before releasing to environmental systems, in the point of view, we have successfully conducted the *in vivo* consequences of oral administration and histological characterization of the heart tissues due to Fe_2_O_3_ (MONPs) has not been documented and identified before and attempt has been made to characterize the possible histological alterations in the heart tissues after oral administration of (MONPs). Additionally, we have focused on exploiting and utilizing Fe_2_O_3_ for biocompatibility studies of heart tissue of albino Wistar rat and melanoma cells (B16-F10), human embryonic kidney (HEK), 293 cells, and *Escherichia coli*, (MTCC 7410) bacterial cell wall damage studies were conducted to synthesized material action (Fig. 1).

## 2 Experimental

### 2.1. Hydrothermal preparation of Fe_2_O_3_

The rhombohedral Fe_2_O_3_ were synthesized by microwave assisted hydrothermal method with slight modification in our earlier report,^25^ to get the perfect rhombohedral geometry, in the point of assessment of toxicity in various biological experiments. FeCl_3_ and NaOH, Ammonia (Sigma Aldrich, India) 10.14 g (37.5 mol) FeCl_3_·6H_2_O and 7.45 g (37.5 mmol) FeCl_2_·4H_2_O were dissolved into 25 ml of H_2_O. 25 ml of 30% liquid ammonia was slowly added to the salt solution under stirring condition at 1000 rpm for 5 minutes, after 20 ml of combination was put into a Teflon-lined stainless Morey autoclave, and the autoclave be heated to 180 °C in an oven and maintained at 10 hour reaction time. Autoclave was naturally cooled to room temperature; the end products were dried under conventional microwave unit at 60 °C and material is ready for characterization.
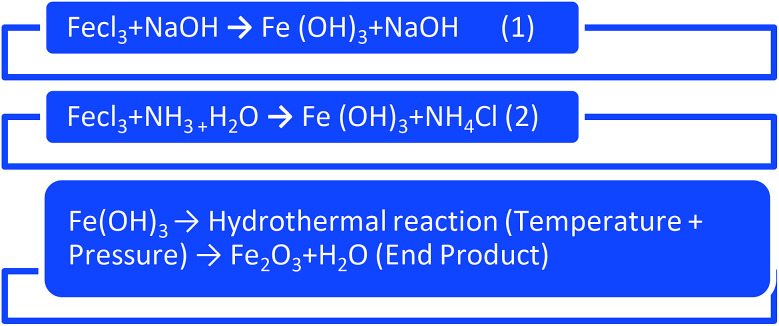


### 2.2. Photocatalytic experiments of degradation of dyes

The photocatalytic behaviour of the synthesized rhombohedral Fe_2_O_3_ were evaluated by the degradation of rhodamine-B (RB) dyes with slight modification to increase the photocatalytic activity. The experiment performed and the catalytic reading taken with the help of Heber-immersion type photoreactor.^35^ In this experiment a specifically weighted amount of photocatalysts was systematically added in the flask with adjusting pH with NaOH and HCl in the similar type of flask at pH 2 to 7. The flask was sited under Heber-immersion type photoreactor, by steady shaking by magnetic stirrer. The experimental trials were performed with an initial rhodamine-B (RB-B) concentration of 9 mM, catalyst concentration of 0.75 g L^−1^.

### 2.3. Animal and treatment studies

An adult male Wistar rats (weight nearly about 130–160 g) were systematically used for the study. The animals were kept in inox steel cages and maintained in a 22 ± 2 °C, 50–80% relative humidity area with 12 hour light/dark pulse. The rats were maintained on a commercial pellet diet and allowed to access deionized water and libitum. All animals were cared for according to the standards of the Guide for the Care and Use of Laboratory Animals. Body weight, water intake, and dietary consumption were monitored daily. After one-week acclimation, the rats were randomly divided into two groups: a control group (*n* = 10), a Fe_2_O_3_ (MONPs) treated group (*n* = 10). Oral administrations of Fe_2_O_3_ nanoparticle were given at dose of 20 mg kg^−1^ respectively,^36,37^ the control rat treated with physiological saline. After 3, 6, 12, 24 and 36 hours treated animals were sacrificed by cervical dislocation. From each animal's heart tissue was studied, the rat tissue was preserved in Bouin's fixative and tissues blocks were embedded with paraffin and sliced into 5 μm thick section, then stained using haematoxylin and eosin.^38^

### 2.4. Determination of anti-bacterial activity and live and dead cell analysis

The Fe_2_O_3_ for bacterial screening along with the control by disc diffusion method,^39,40^*Escherichia coli*, (MTCC 7410) bacteria. Hydrothermally synthesized Fe_2_O_3_and control were prepared in sterile distilled water (stock solution) over a range of different concentrations in 100 mg ml^−1^. The analysis was performed to distinguish dead and viable bacterial cells upon treatment with Fe_2_O with minor modifications.

### 2.5. B16-F10 cell culture and treatment and normal cell line toxicity testing using HEK-293

The B16-F10 cell culture dose-dependent response curve was plotted using propagation and MTT [(2-(3,5-diphenyltetrazol-2-ium-2-yl)-4,5-dimethyl-1,3-thiazole bromide)] assay, after 72 hours of exposure was determined according to,^41,42^ HEK-293 (human embryonic kidney), cell lines were gotten from the American Type Culture Collection (ACC), USA. The methodology involved in HEK-293 cell line IC_50_ assessment, parallelly the test Fe_2_O_3_ nanomaterial inhibits the 50% of the proliferating the normal cells as per above environmental conditions for 48 hours.^43,44^ This procedure is non-hazardous, uses a thermally stable reagent and shows good correlation with constant proportion.^45^ The experiments were performed in triplicate and three independent repetitions.

### 2.6. Characterizations and measurements

The structure and composition of Fe_2_O_3_ (MONPs) were characterized by a various tools; Brunauer–Emmett–Teller (BET) specific surface area, pore volume, and pore size distribution of the samples were determined by N_2_ sorption at 77 K using a Micromeritics ASAP-2020, Ultima-III Series, RIGAKU, TSX System, Japan, was used to assess the crystallinity with wide-angle X-ray diffraction (XRD) patterns, high resolution transmission electron microscopic (HR-TEM) images of Fe_2_O_3_ (MONPs) was captured using Jeol/JEM 2100, LaB_6_, 80–2000 mm with 200 kV acceleration voltages. The tissue section examined with the help of Carl Zeiss, Axio image A_2_M, advanced polarizing microscope, USA. The Energy Dispersive Spectroscopy (EDS) analysis was carried out using HITACHI (Noran System 7, USA) system attached to the Zeiss Supra 55VP, FE-SEM is an extremely soaring resolution field emission scanning electron microscope for the detection of composite nanoparticles. Fourier-transformed infrared (FT-IR) spectra were obtained on a Thermo Scientific Nicolet, 6700 Analytical FT-IR spectrometers. Zeta potential measurements of the attenuate dispersions (0.1 mg mL^−1^) of the nanocomposites were conducted using a Brookhaven Nano-Brook Omni Instrument at 25 °C. The particle size distribution (PDS) nanocomposite was monitored by using Microtrac (USA) particle size analyser. The analyser provides the size measurement, which confirms the particle size distribution. The measurement of the specific surface area of materials by using Brunauer–Emmett–Teller (BET), BEL:2 SORP, Japan and atomic force microscopy (AFM), APE Research, AFM, 100, ITALY used to determine the resolution on the order of fractions of a nanometre of a synthesized material. The photoluminescence results were taken from photoluminescence spectrometer, FLS-1000, the identification of rhodamine-B (RB) degradation intermediate products were measured by a liquid chromatography-mass spectrometer (LC-MS) model, (Waters-USA, Synapt G-2 HDMS). Microscopic with camera Olympus, (USA, 2000) used to take the photograph of HEK, 293 control and effected cell lines. The responding data replicates (three) were analysed for every attempt and for every analysis of discrepancy (ANOVA) using SPSS-Inc. 22.0. Trivial effects of treatments were resolved by *F* values (*p* ≤ 0.05).

## 3 Results and discussions

### 3.1. Characterization results of synthesized rhombohedral Fe_2_O_3_ nanomaterial

**Fig. 2:**
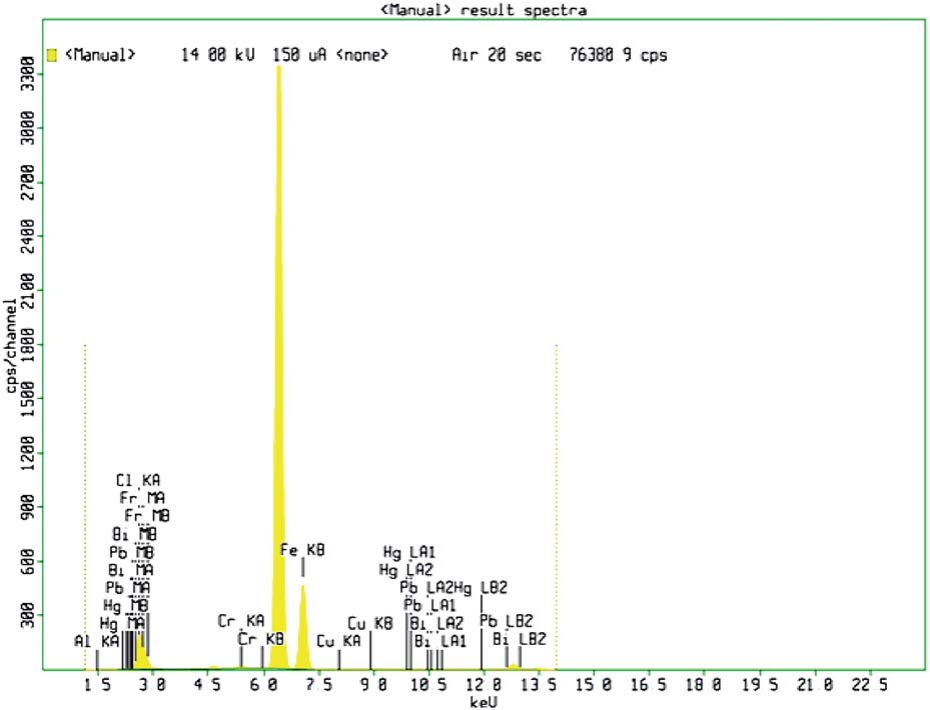
X-ray fluorescence spectra of Fe_2_O_3_ metal oxide nanoparticle.

The X-ray fluorescence is recorded with fine focus X-ray tube, MO target of multi-layer monochromator of 17.5 keV.^46^ The XRF spectra of Fe_2_O_3_ clearly indicate that the presence of Fe^3+^. XRF pattern of Fe_2_O_3_ nanomaterial are shown in Fig. 2. The diffraction peaks in 6.2 to 7.2 keV and the concentration of the compound of Fe-KA is 153 256.1 cps are perfectly aligned to the rhombohedral phase in Fe_2_O_3_.

**Fig. 3:**
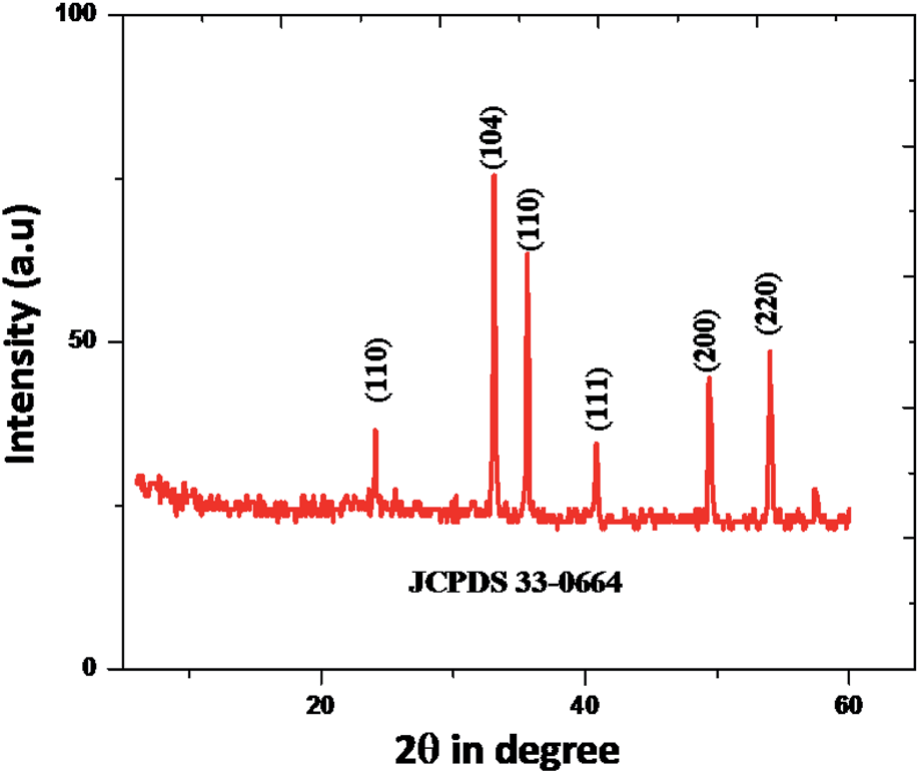
X-ray diffraction profile of synthesized Fe_2_O_3_ nanoparticle.

In Fig. 3, shows XRD patterns of the samples obtained at 180 °C in 10 hour hydrothermal reaction. According to the figure, it clearly shows that, the position of the diffraction peaks is in a good argument with those of rhombohedral Fe_2_O_3_ (JCPDS no. 33-0664).^47,48^ There is no other crystalline and impurities, those results clear indication of purity of the nanomaterial. However, the diffraction peaks have shown a clear geometric shape in the point of utilization in various applications. The mean crystallite sizes of Fe_2_O_3_ from the XRD data effectively by using Debye–Scherer formula. The mean crystallite sizes of rhombohedral Fe_2_O_3_ is 10 nm, An HR-TEM and EDX result gives a clear justification to this argument.

The properties of Nano-structured materials habitually depend on their morphology and size. Table S1 in ESI† shows FT-IR spectrums of Fe_2_O_3_ nanoparticles in the range 400–4000 cm^−1^, nanoparticles show the spectral absorption in 3420, 3192, 1603, 1388, 1114, and 454 cm^−1^. The absorption peaks at 3420 and 1603 cm^−1^ can be assigned to the stretching vibration and bending vibration of OH groups of H_2_O.^49^ The bands at 1114 and 454 cm^−1^ can be attributed to the Fe–O stretching vibration modes in Fe_2_O_3_. This FTIR results indicate the formation of Fe_2_O_3_ in the present outcome, which are reliably matched with XRD patterns. It is evident that, the FTIR peaks of Fe_2_O_3_ nanoparticles have higher frequency to compare with already reported morphologies.^50,51^

**Fig. 4:**
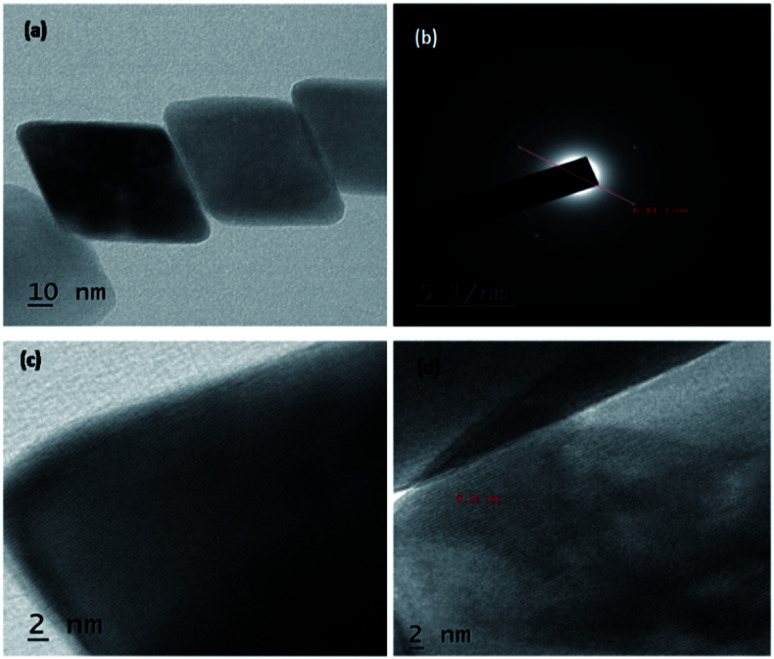
HR-TEM images of Fe_2_O_3_ nanoparticles, (a) rhombohedral Fe_2_O_3_, (b) SAED of Fe_2_O_3_ material (c) and (d) nanomaterial selected area of electron diffraction (SAED) pattern and magnified area of region.

High resolution-TEM of synthesized rhombohedral shaped Fe_2_O_3_ nanoparticles was shown in Fig. 4; from the images it evidently showed that, the high-quality geometry, in addition, the material results in the formation of mesopores between the particles (Fig. S6 in ESI†), which clearly confirmed by BET surface area and pore volume measurements. The size of both the individual nanoparticles was between 10 to 20 nm which are in concordant with the results of XRD, DLS and also FT-IR, and those results are in a good argument with Debye–Scherer formula in the above stated results. It was also observed that, the *d*-spacing measurements of twin domains were measured to be approximately 0.2 Å to 0.4 Å nm and corresponding to (110) to (220) planes of Fe_2_O_3_, respectively, the interplanar spacing of ∼0.25 nm agrees well with the spacing between (110) planes of rhombohedral iron oxide crystals (0.25 nm) and SAED patterns are the good proof for compact arrangement of the nanoparticles.

**Fig. 5:**
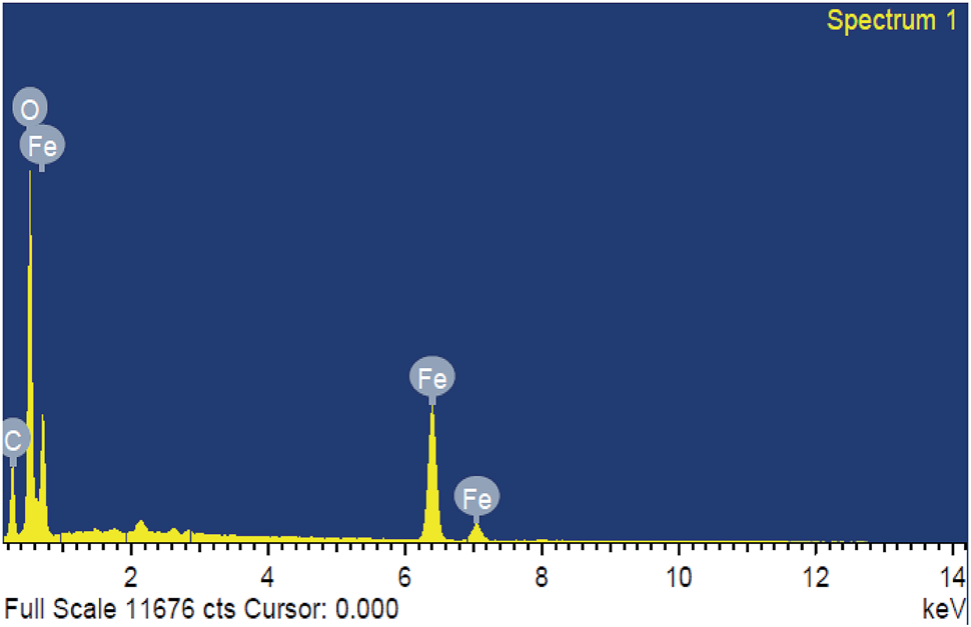
The energy dispersive spectrum elemental composition of Fe_2_O_3_.

Energy dispersive spectroscopy (EDS) analysis confirmed the phase transparency of the Fe_2_O_3_ as shown in Fig. 5. The characteristic peaks of Fe and O appear in the spectrum of Fe_2_O_3_ and confirming its successive formation and purity (Table S1 and Fig. S7 in ESI†). The peak of (C) carbon seems due to the carbon tape used sample container in the energy dispersive unit. The particles histogram using DLS of Fe_2_O_3_, distribution of particle sizes when immersed in the solvent have a range from 10–20 nm. Zeta potential measurement is the reliable method for assessing material interaction with other biological system. The particles examined presently possessed a zeta potential for Fe_2_O_3_ as – 13.9 mV (Fig. S6 and S7 in ESI†), indicating safe to environmental as well as biological utilization.

**Fig. 6:**
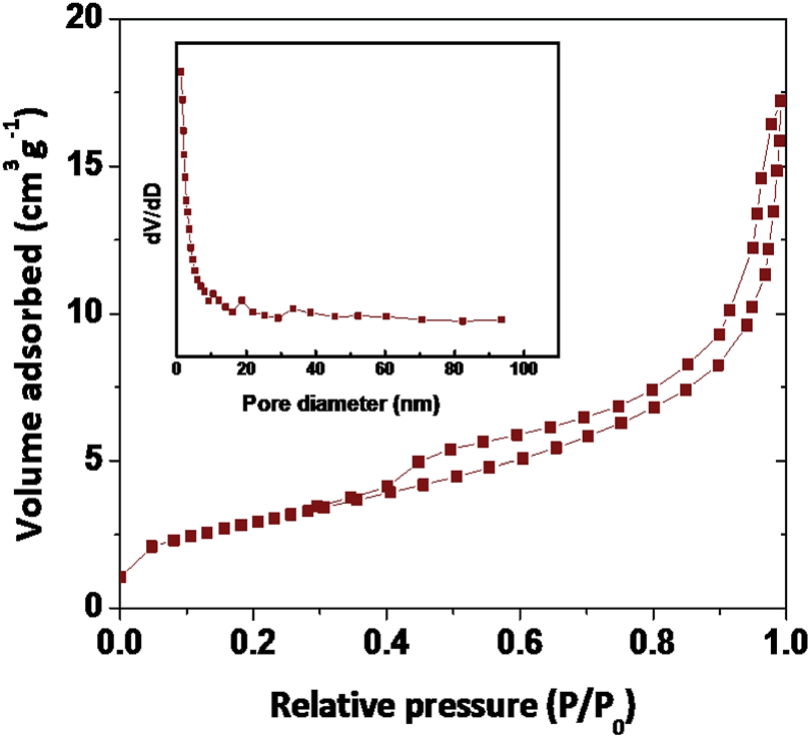
The BET N_2_ absorption/desorption graph of rhombohedral Fe_2_O_3_.

The surface area is an important tool to determine the biological adoptability of nanoparticles and nanomaterial with a high surface area is easily attracting the biological material at the faster rate due to his inert nature. The superior specific surface area provides more surface-active sites and well-organized transport lanes to reactant molecules and products in environment as well in biological systems. BET surface area as a function of pore volume and the size of the synthesized samples present in similar (type-II).^10,52,53^ The BET surface areas reading of Fe_2_O_3_ is 5.676 m^2^ g^−1^ (Fig. 6 and Table S2 in ESI†). According to the hysteresis loop in the relative pressure region approximately 0.4–0.9, the nitrogen adsorption/desorption isotherms showed that the Fe_2_O_3_ exhibited a similar (type-IV) curve and it is in nanoporous structure, with an increase in the surface area and decrease in the pore size, as well as the large total pore volume, of Fe_2_O_3_ is expected to have a high biological hold up.

**Fig. 7:**
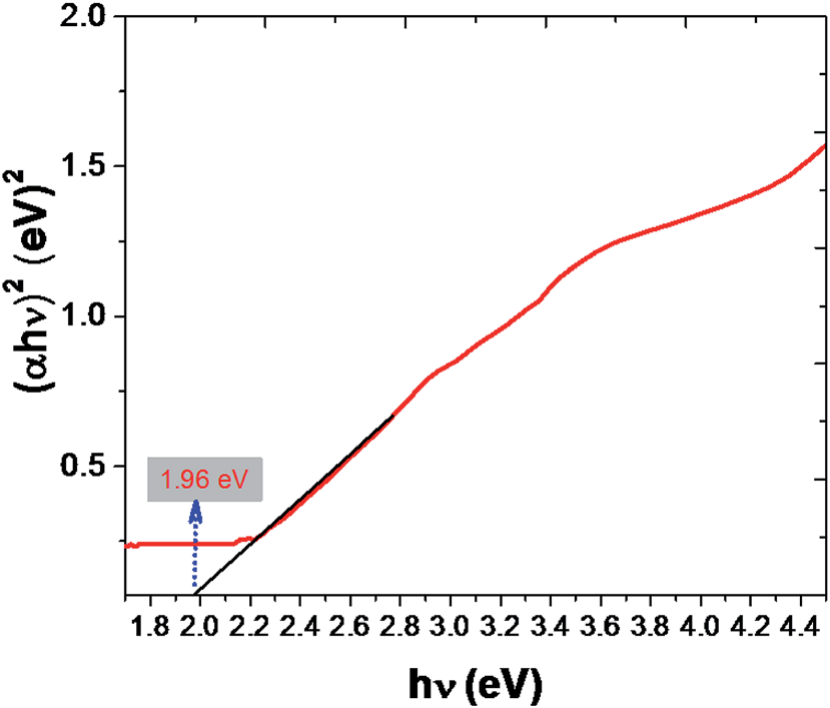
The Fe_2_O_3_ band-gap calculation by using Tauc plot.

UV-Vis diffuse reflectance spectra (DRS) and band-gap energy of Fe_2_O_3_ are shown in Fig. 7. DRS were used to investigate the light-harvesting nature of the Fe_2_O_3_ photocatalyst, but in this study we are effectively adopting to determine the material environment for biological susceptibility. Conduction-band minimum (CBM) and valence-band maximum (VBM) is play a vital understanding to mechanism absorption on biological materials. To investigate the CBM and VBM of the Fe_2_O_3_. UV-DRS spectra were used to record the spectrum. The associated band-gap values were calculated using the equation in ref. 54. Which are consistent with the similar results obtained from the related work.^55,56^ Evidently calculated band-gaps of rhombohedral Fe_2_O_3_ were found to be 1.96, this reading is important, because of nanomaterial can be photo excited to generate more electron–hole pair under visible-light irradiation, it's results have higher catalytic performance. This mechanism leads to more impact on natal material effect (Table S3 in ESI†).

**Fig. 8:**
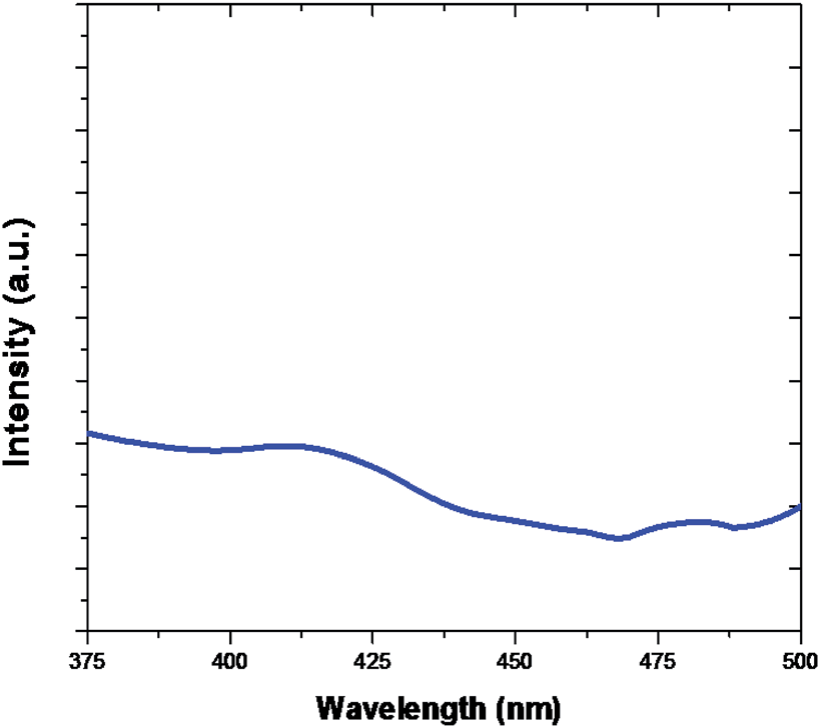
Photoluminescence (PL) spectra Fe_2_O_3_.

PL spectra of Fe_2_O_3_ are evidently shown in Fig. 8. The PL emission is light source emission from any form of matter after the absorption of photons. The peaks are observed at 416 nm and 440 nm. In general photoluminescence positive energy can be viewed as an indication of electron populated an exited state associated with transition energy.^56–58^ The true atoms are the similar systems, correlations and many more multifaceted trends and also act as starting place for photoluminescence in catalytic and also many body systems.

The atomic force microscopy (AFM) image shows synthesized material in a high quality, it is essential to execute corrections of the sample slope and background.^59,60^ Apropos the instabilities occurred during scanning, especially in the ranges under 4 μm, the AFM used to assess the particle size and morphology and thus to explain the colour difference. The iron oxide nanoparticles with the size of 10–20 nm and with the morphology corresponding to very thin rhombohedral geometric structures, particles of metal oxide nanomaterials prepared by hydrothermal route method, just the different values of vertical dimensions, which are accountable for dissimilar colour excellence, cannot be effortlessly found by using other techniques together with HR-TEM.

**Fig. 9:**
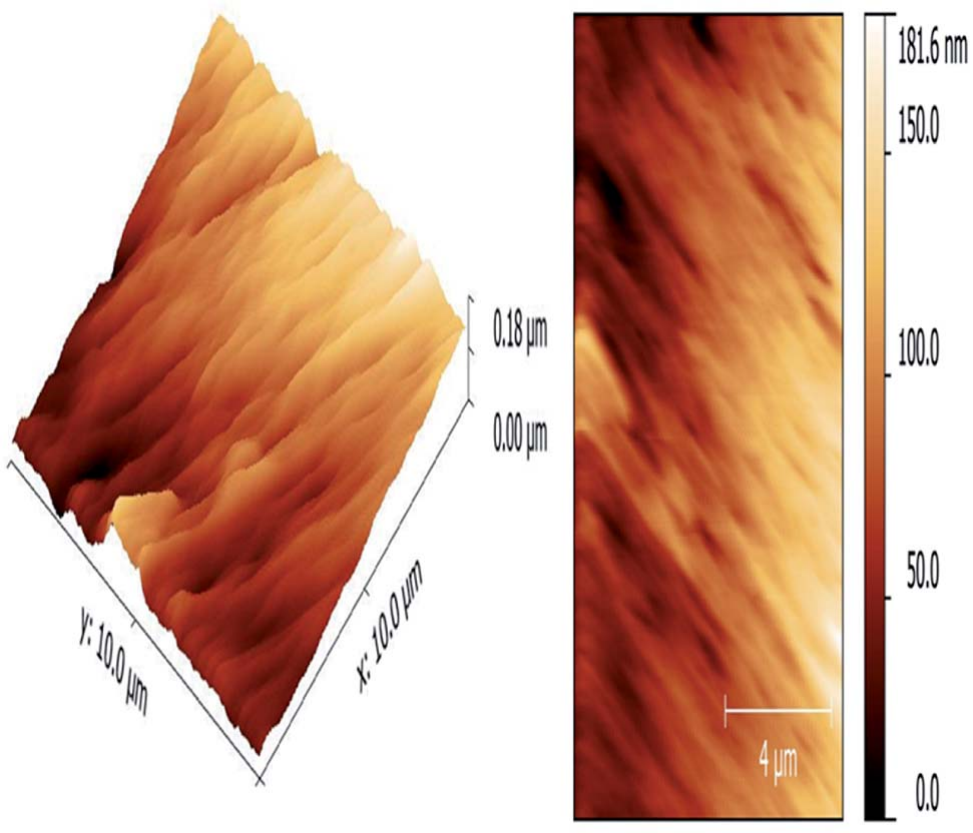
Atomic force microscopy (AFM) for the characterization of Fe_2_O_3_ rhombohedral nanomaterial.

Surface roughness (*R*_a_) value, the *R*_a_ value indicates average of a set of individual measurements of a surface's peaks or valleys. The *R*_a_ value for rhombohedral Fe_2_O_3_ was 27.2 nm that shows the evenness of nanomaterial (Fig. 9). Due to rough surface texture of synthesized nanoparticle, there was well absorption in heart muscles caused inflammation like increased neutrophil count was observed in this study.

In the photocatalytic activity of nanomaterials effect of pH play a vital role.^61^ The forethought of rhodamine-B (RB-B) (Fig. S9 in ESI†) and catalyst dosage were fixed at 9 mM rhodamine-B (RB-B) and 0.75 g L^−1^ (rhombohedral Fe_2_O_3_), respectively. The outcome of pH on the photodegradation of rhodamine-B (RB-B) is revealed in Fig. S2 in ESI†. pH zero-point charge (zpc), the external play an important role in dye degradation and mineralization studies.^62,63^ The zpc values of rhombohedral Fe_2_O_3_ is 7.4 respectively, this clearly below pH the surface of material is positively charge and dye was simultaneously degraded and nontoxic small molecular intermediates was obtained (Table S7 in ESI†).

The photocatalytic activity of ZnO, TiO_2_ and Cu_2_O for clear determination synthesized nanomaterial behaviour. This research confirmed that 82.14% of rhodamine-B (RB-B) was besmirched by the Fe_2_O_3_ nanomaterials at 120 minutes of irradiation time in Heber-immersion type photoreactor. The degradation percentages of commercially available ZnO, TiO_2_, and Cu_2_O are 59.61%, 69.87%, and 70.56%, respectively. The solar induced photocatalytic activity of rhombohedral Fe_2_O_3_ (97.42%) is advanced when compared to the standards in Fig. S3 in ESI†.

**Fig. 10:**
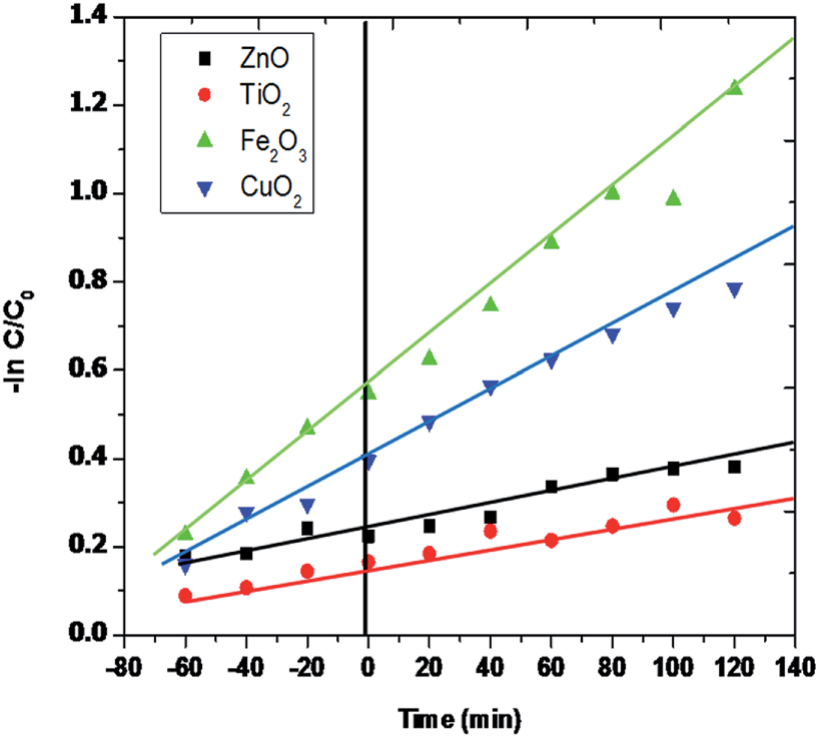
Photocatalytic degradation kinetics of rhodamine-B (RB-B) using various photocatalysts.

The Langmuir–Hinshelwood model.^12,64^ was efficiently used to figure out the degradation kinetics of rhombohedral Fe_2_O_3_ photodegradation. Fig. 10 explains the apparent logarithmic plot of rhodamine-B (RB-B) concentration as a utility of irradiation time. The pseudo-first-order rate kinetics explains Fe_2_O_3_ is 2.65 × 10^−2^ s^−1^ is appreciably higher than of TiO_2_ (5.12 × 10^−3^ s^−1^) and CuO_2_ (7.38 × 10^−3^ s^−1^). For this reason, the activity of the Fe_2_O_3_ rhombohedral material is about 2.7 percent superior than that of other examined material and the ZnO (2.01 × 10^−3^ S^−1^) shows the trifling photocatalytic activity to compare to other efficient materials in various studies so far.

**Fig. 11:**
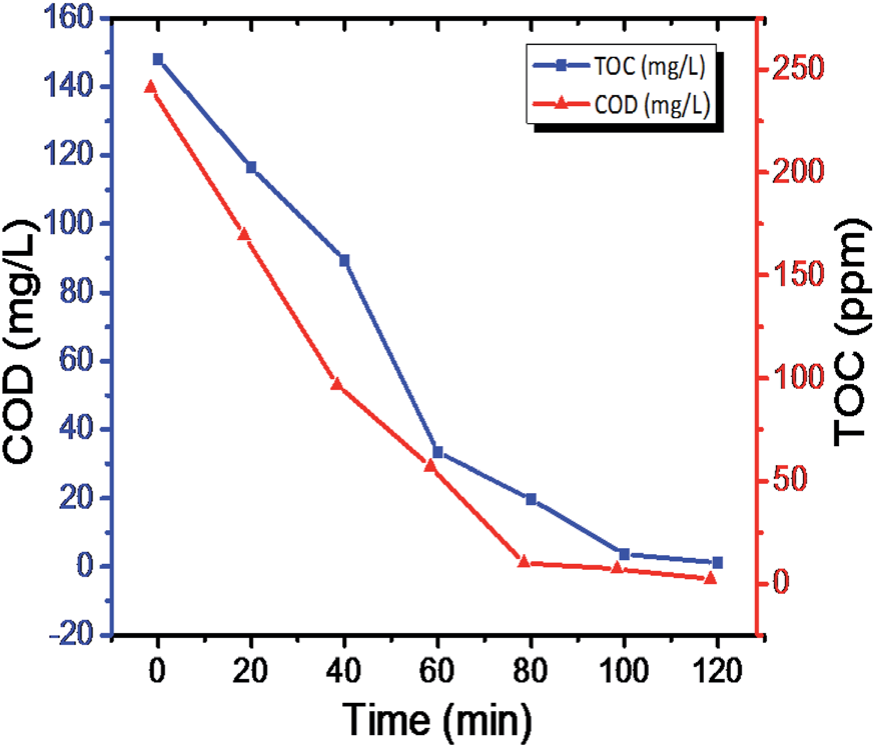
Chemical oxygen demand (COD) and total organic carbon (TOC) readings.

The photodegradation intermediates in the reaction process were help to predict the actual reaction process.^65,66^ The tiny molecules during mineralization process are responsible for CO_2_ and H_2_O, the COD and TOC (Table S4 in ESI†), results evident to photodegradation process (Fig. 11). The two spirited actions have occurred at the same time during the photoreaction: *N*-deethylation and destruction of dye chromophore structure,^67^ The negative correlation was observed in COD and TOC in respective time consider for the study of degradation (Fig. S9 and S10 in ESI†). Interpermeates of photocatalytic degradation of rhodamine-B (RB) were performed using LC-MS and results were hypothesized in ESI (Table S7 in ESI†).

**Fig. 12:**
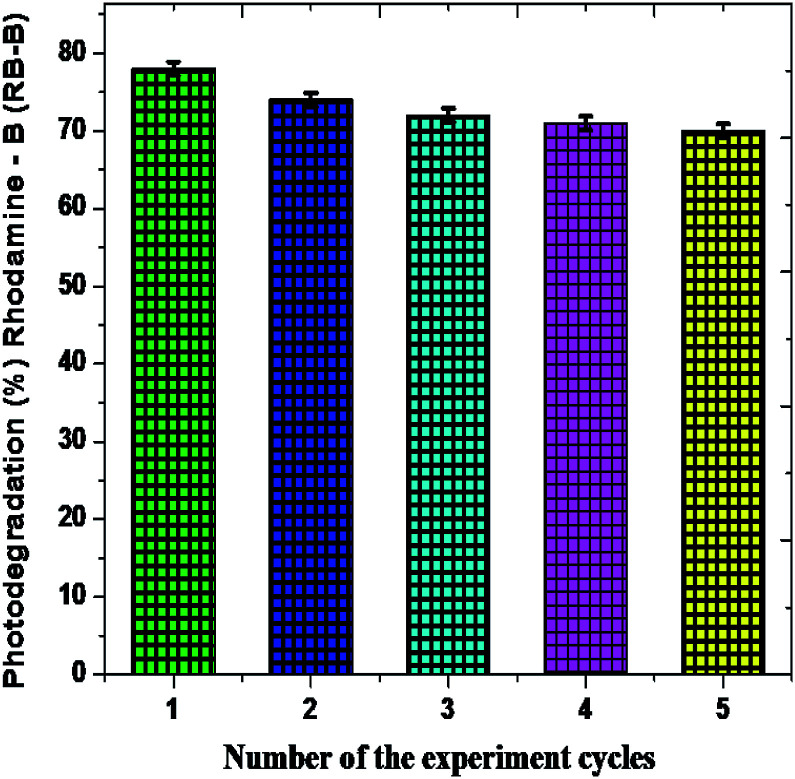
Rhombohedral Fe_2_O_3_ catalyst recycling performance cycles.

The recovery of a photocatalytic nanomaterial is the most important for eco-friendly utilization of product.^68,69^ In order to examine the constancy and stability of the Fe_2_O_3_ nanomaterial is essential after the photodegradation of rhodamine-B (RB-B). The photodegradation proportion of five consecutive cycles is 76.23%, 73.78%, 72.56%, 71.09% and 71.43%, respectively, and showed in Fig. 12.

**Fig. 13:**
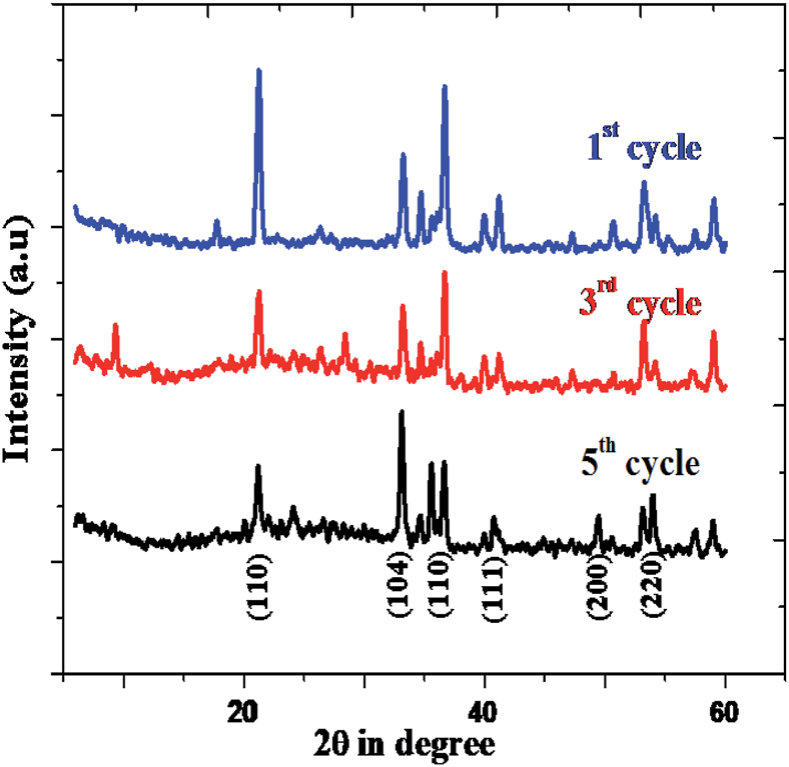
XRD pattern rhombohedral Fe_2_O_3_ nanoparticle.

The rhombohedral Fe_2_O_3_ nanomaterial exhibits an average of 73.18% of solar driven activity after five successive cycles. In addition, there is slight change observed in the XRD pattern of Fe_2_O_3_, due to absorption of small molecular intermediates organic material present in dye (Fig. 13), after five cycles.

**Fig. 14:**
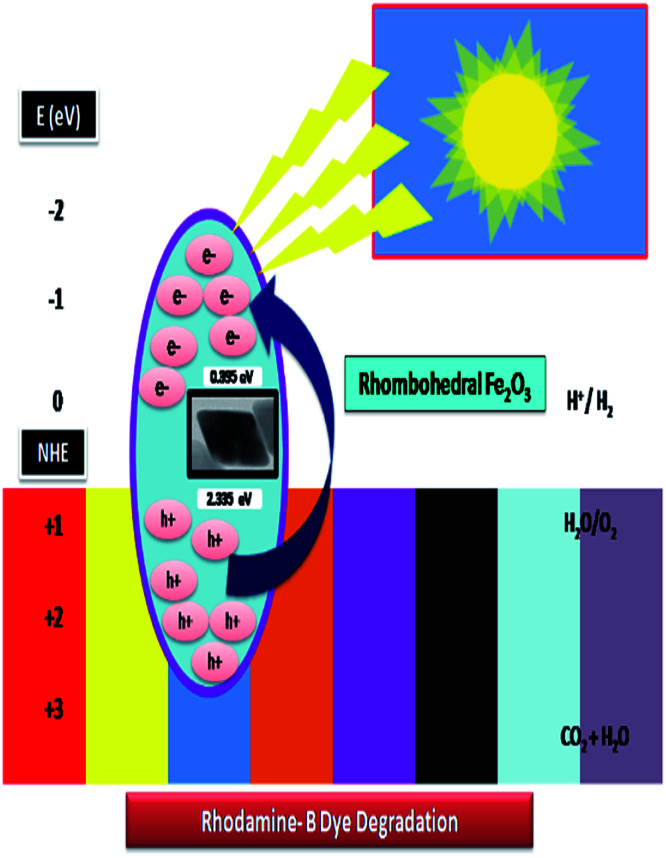
Probable photocatalytic dye degradation mechanism *via* visible light irradiation.

The systematic hydrogen production by photo-induced water break-down was more captivating and give a suitable solution for energy conversion and conversional problems.^65,70^ H_2_ generation methods and photocatalytic water excruciating using visible light spectrum with low cost and more sustainability features in the photo reaction system are more popular. This radical piercing process makes a crucial role in the degradation of dyes rhodamine-B (RB-B) concentration. In our experiment, we are proposed the photo-reaction efficiency of the adsorption of untreated organic dye on the by rhombohedral Fe_2_O_3_ and the progression of splitting photo-twisted electron–hole pairs. The holes can either react through rhombohedral pore surface, and form hydroxyl to hydroxy radicals. Consequently, the degradation was occurred to allowing dye molecules to move from solitary explication to the material interface and to subsequently carbon dioxide (CO_2_), water (H_2_O) and other small molecular intermediates are the by-products of redox reactions as clearly showed in Fig. 14.

### 3.2. Biological toxicity results of synthesized rhombohedral Fe_2_O_3_ nanomaterial

**Fig. 15:**
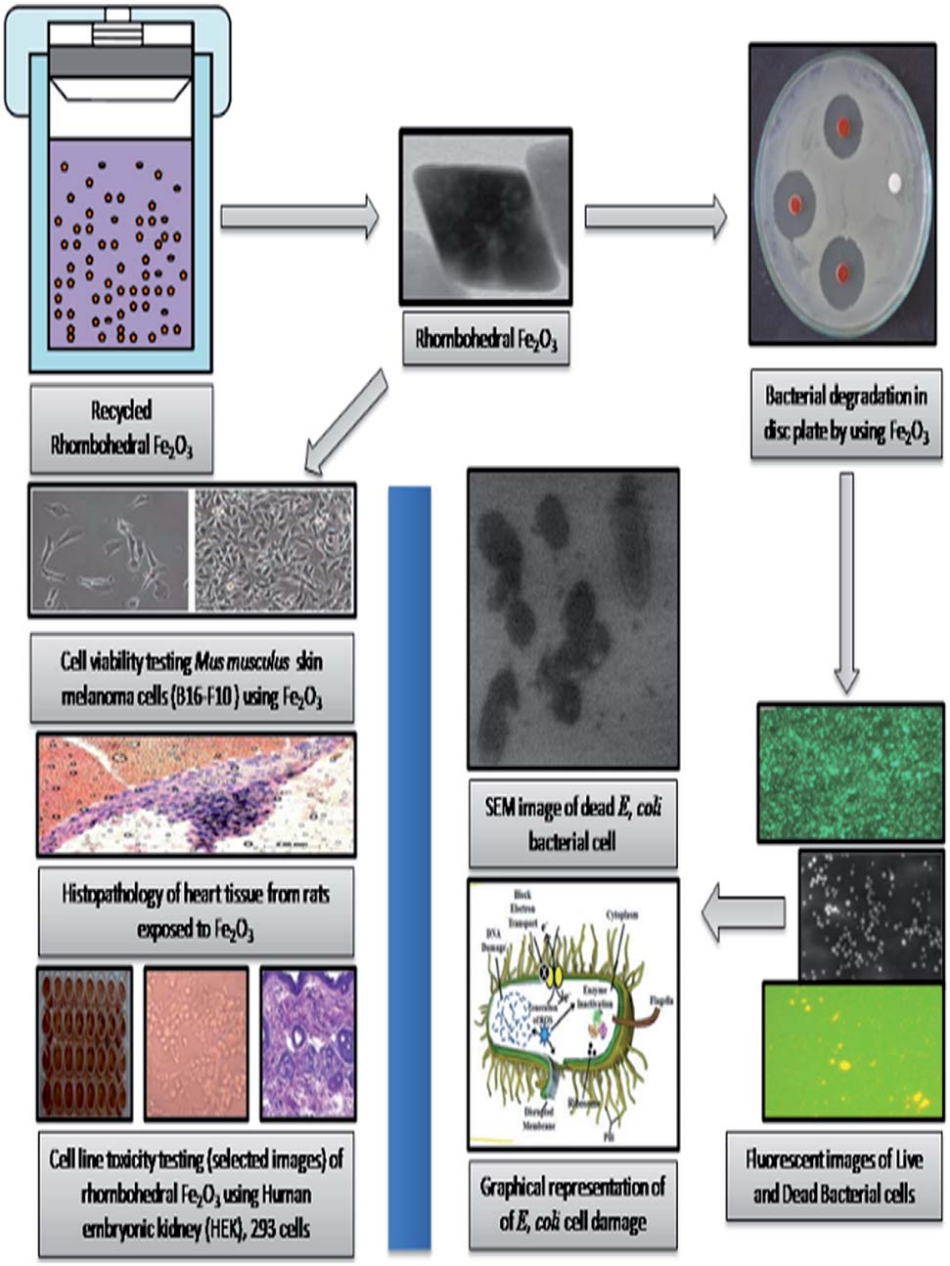
Toxicity assessments using rhombohedral Fe_2_O_3_.

The epidemiological studies have clearly recommended that, health effects of nano and micro particles dependent one and each other.^71,72^ An earlier toxicity studies showed that nanoparticle was made specific acute effects while micro particles induced chronic effects.^73–75^ In this study, our study explains the environmental and health concern by using biological specimens mimic oral exposure. We found that structured Fe_2_O_3_ nanoparticles entered the body *via* oral administration; they became systemically and cause toxic effects in cardiovascular system. These actions were measured to be the essential mechanisms of organ damage. The metal oxide nanoparticles (MONPs), due to his high calibre in their size, they are probably transported from the oral way into body circulation, and reach cardiovascular system and made remarkable hazard. Metal oxide nanoparticles (MONP's) by products from industrial as well as biomedical output will in aquatic systems. However, some studies have shown that the solubility of metal oxide nanoparticles in biological liquids is in addition one of the factor that, determines legatee velocity of penetration into blood streams,^76^ especially Fe_2_O_3_ nanoparticles has been shown to dissolve in normal environment and easily get in to living systems.^77–79^ Simultaneously, *musculus* skin melanoma cells (B16-F10), human embryonic kidney (HEK), 293 cells, and *Escherichia coli*, (MTCC 7410) bacterial cell wall impairment also carryout (Fig. 15).

**Fig. 16:**
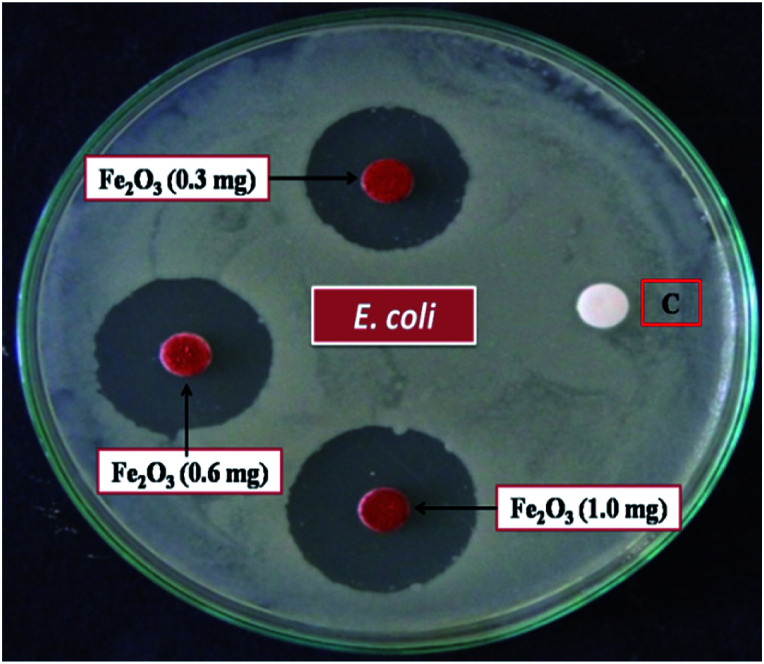
Anti-bacterial activity of *Escherichia coli*, (MTCC 7410) treated against different concentration of rhombohedral Fe_2_O_3_ material *via*, disc diffusion method, [C] sterile distilled water.

**Fig. 17:**
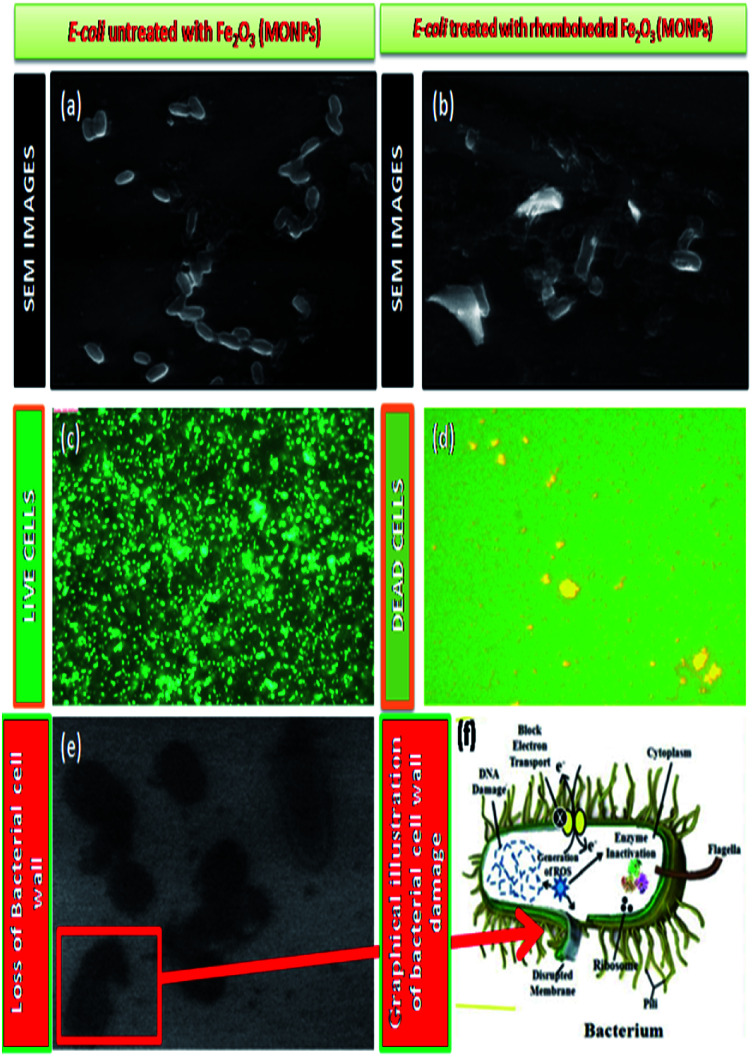
Scanning Electron Microscopic (SEM) images of (a) *E. coli*, (Live cells) untreated with Fe_2_O_3_, (b) *E. coli*, (Dead cells) treated with Fe_2_O_3_, (c) and (d) selected live and dead cell representative fluorescent microscopic images (40×), Fe_2_O_3_ in general both the series, green dots represent live bacterial cells and yellow/orange dots represent dead cells. (e) *E. coli*, cell wall loss by Fe_2_O_3_, (f) graphical representation of cell wall damage.

The result of anti-bacterial activity showed that, there exists a significant zone of inhibition against test pathogens, many studies have proved that the photo-generated holes and ·OH are the main oxidative agents for *Escherichia coli* suppression. It should be noted that, the test samples, due to its large surface area Fe_2_O_3_ nanoparticle, *Escherichia coli*, (MTCC 7410) were used for study with different concentrations of material were added to the disc plate containing a nutrient agar media (NA), yields the maximum inhibition zones 20.60 ± 0.24 at 1.0 mg, followed by 0.6 mg is 18.35 ± 0.08, and 0.3 mg was 15.81 ± 0.15 (Table S5 in ESI†), respectively (Fig. 16). We did not observe any zone of inhibition in SDW (Sterile Distilled Water) diffused discs against test pathogens. This revise clearly suggests that, Fe_2_O_3_ material inhibit the *E. Coli* (MTCC 7410) bacterial pathogens by rupturing the outer and inner wall of the cell which leads to toxic and damage the cell membrane. The fluorescent microscopic images (Fig. 17) are agreed the clear evident of bacterial death.

**Fig. 18:**
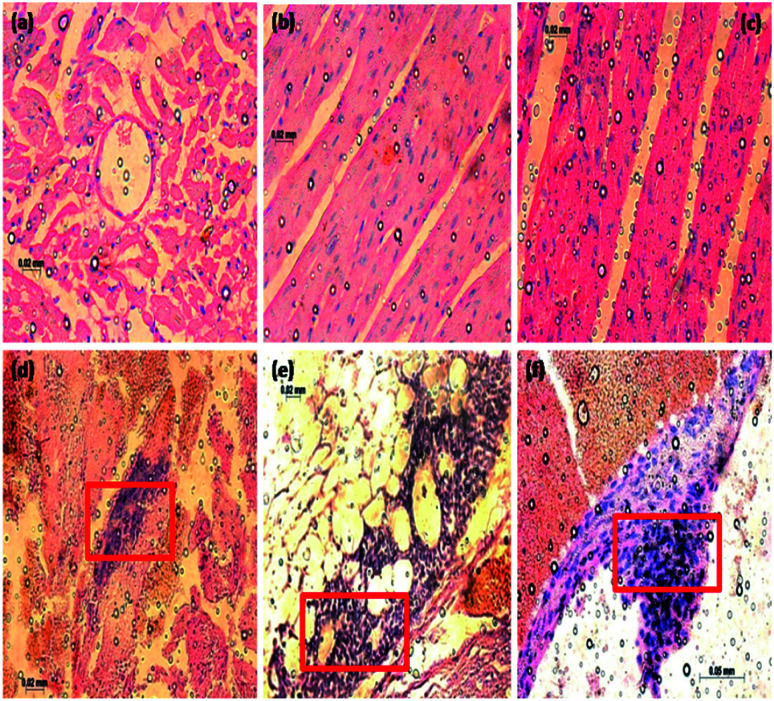
Histopathology alterations of heart tissue from rats exposed to Fe_2_O_3_ nanoparticles for 3 days (magnification = 0.2–0.5 mm). The rats were sacrificed at 3, 6, 12, 24 and 36 hours after the final administration. (a) No inflammation in control group, (b) Fe_2_O_3_ treated rats at 3 hours with no toxic effects observed in cardiac tissue, (c) Fe_2_O_3_ treated rats at 6 hours with minimal inflammation were observed, (d) Fe_2_O_3_ treated rats at 12 hours with mild neutrophilic infiltration on cardiac muscle due to the time interval, (e) and (f) Fe_2_O_3_ treated rats at 24 and 36 hours shows a maximum neutrophilic infiltration was observed.

In Fig. 18a, normal rat demonstrating normal heart muscle, the rhombohedral Fe_2_O_3_ treated rat received 20 mg kg^−1^ of 10 nm particles for 0 to 6 hours (Fig. 18b and c) demonstrating benign normal looking heart muscle with normal muscle direction and fascicles with no pathological effects. The metal oxide nanoparticles (MONP's) for 6 to 12 hours (Fig. 18d and e), demonstrating spread in the extravasations of red blood cells, packed dilated blood vessels, prominent focus of tiny lymphocytic infiltrate associated with focus of muscle hyalinosis, troubled muscle fascicles and scattered red blood cells. In the view of 24 to 36 hours (Fig. 18f), demonstrating dense prominent focuses of inflammatory cells infiltrate of minute lymphocytes and few plasma cells and prominent congested dilated, blood vessels and few scattered extravasation red blood cells. Histological studies induced in the heart tissue exposure could be an indication of congested cardiovascular system with extravasations, red blood cells and vessels, heart muscle hyalinises, troubled muscle fascicles, inflammatory cells infiltrate by small lymphocytes and plasma cells, due to higher dose Fe_2_O_3_ toxicity. The nano residue accumulated heart muscle leads to metabolic and structural changes. The increased neutrophil counts have been indicated in the red coloured square boxes in the respective images.

**Fig. 19:**
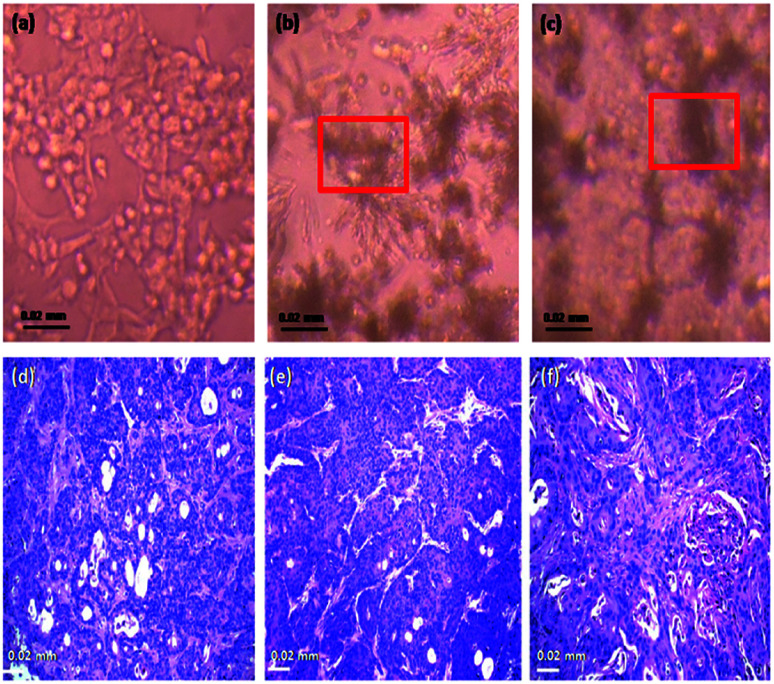
Cell line toxicity testing (selected images) of rhombohedral Fe_2_O_3_ using human embryonic kidney (HEK), 293 cells, (a) and (d) control cell line, (b) and (e) human embryonic kidney (HEK), 293 cells, treated 300 μg ml^−1^ of nanomaterial showed in-site box and (c) and (f) accumulated material were observed in 500 μg ml^−1^ treated with respective cell line. The red colour boxes in the (d) and (c) shows the nanoparticle deposition and cell damage.

The projecting anti-proliferative outcome of functionalized Fe_2_O_3_ on HEK-293 as exposed by its IC_50_ built on XTT assay was instigate to be 202.14 ± 0.14 equated with the standard drug cisplatin >500 (Table S6 and Fig. S8 in ESI†). Consequently, the hydrothermally synthesized material is the promising aspirant for hopeful drug to anti-cancer with its toxicity. However, the synthesis methodology gives a nontoxic nature to rhombohedral Fe_2_O_3_. The plasma semi-penetrable membrane allows freely dissemination of tiny and non-polar molecules available in the synthesized material were uptake the endocytosis. Most of chemically worked Fe_2_O_3_ are easily taken up by the cells through endocytic mechanisms, and persist in endosome vesicles, and become incapable of reaching the cytosol system. The proficiency mainly depends on nanomaterial size, shape, physical, chemical and its dispersive nature. Finally, the hydrothermally synthesized rhombohedral metal oxides can be used effectively at a very low concentration against different types of cell lines for the drug delivery procedures and also, pharmaceutical applications. The efficient permeability effects (EPR) of nanoparticle play an vital role with a solution for frequent toxicity assessment strategies through cell line, the targeting efficiency *via* a wide spectrum of nanomaterial based on various engineering methodologies,^80–83^ up till now, ample of immunotherapy strategies against cell line toxicity have undergone extensive development and clinical testing, from.^84,85^ HEK-293 cells have been extensively utilized in biological research for many years, for the reason of consistent development and penchant. The therapeutic effect of human embryonic kidney (HEK), 293 cell line efficacies by rhombohedral Fe_2_O_3_*in vivo* toxicity effect were inoculated in the right flank of mice and applied treatments 7 days later. The cell line volume was recorded in the following two systematic weeks. Fe_2_O_3_ exhibited the most excellent on cell line growth with was compared with free drugs loaded vessel systematically (Fig. 19a). Obviously, there is effect in the photograph of the normal cell line, the staining pictures of cell line sections from each cluster were dependable with the results like incompact cells, shrunk nuclei and damaged morphology were observed in both (Fig. 19b and c), which reflected more advanced cell apoptosis was induced by Fe_2_O_3_, after systemic administration. We deem the conception of this toxicity combined approach could also be applicable for other proper anti-cancer drugs and immune-stimulating agents to rescue the liposomes in future.

**Fig. 20:**
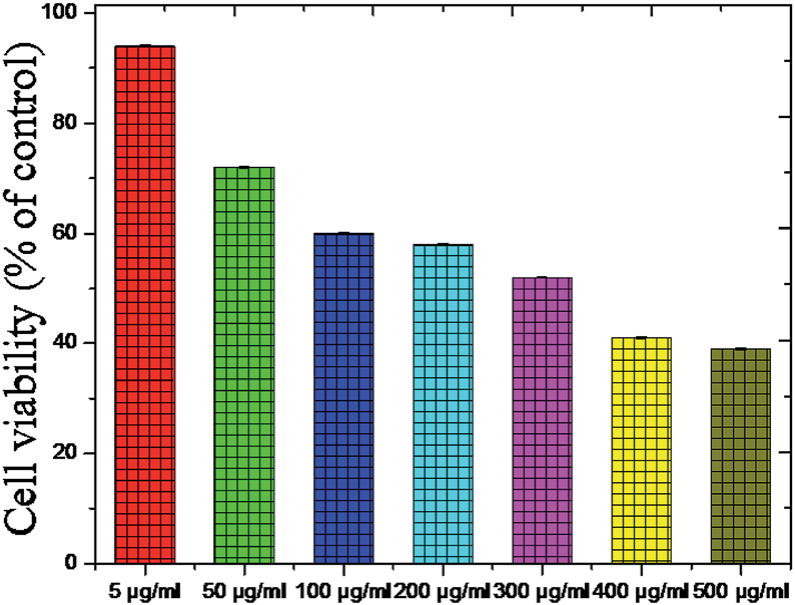
Toxicity testing of the Fe_2_O_3_ material using melanoma cells (B16-F10) cells using various concentrations.

The cell viability is the major biocompatibility tool now a days.^86–89^ We found out the toxicity of the Fe_2_O_3_ using *musculus* skin melanoma cells using numerous concentrations (5–500 g mL^−1^). The results clearly suggested that the viability of B16-F10 cells have decreased in a dose-dependent manner in each of the sample. Fig. 20 clearly showed that, 5 to 50 g mL^−1^ concentration, the lowest concentration of three respective samples used in the assay did not reduce the cell viability noticeably as compared to other increased concentrations, therefore, Fe_2_O_3_ in lower dose is the safest and the superlative alternative in terms of toxicity in biological application.

## 4 Conclusions

In this experimental work, a unique a rhombohedral Fe_2_O_3_ has been prepared by a simple, eco-friendly and low-cost hydrothermal method for multi-purpose application like rhodamine-B (RB-B) and biological toxicity testing by using cardiovascular muscle of rats, human embryonic kidney (HEK), 293 cells, viability cell testing in melanoma cells (B16-F10) and *Escherichia coli*, (MTCC 7410). The lower doses of synthesized material were found to be highly stable, low toxic in nature, in the case of increasing the dose level rhombohedral Fe_2_O_3_ was cause the hazardable toxicity to the various experimental models. Our material gives excellent photocatalytic activity and less toxicity to compare with single, bi and tri-composite nanomaterials. This hydrothermal method synthesis and exposure to biological models tells fate of safe utilization material and the results are hopeful can be utilized these materials to biomedical field and environmental conversion systems.

## Live subject statement

The animal welfare and the experimental procedures were carried out in accordance with the regulations for the administration of affairs concerning experimental animals, and the Ethical Regulations on the care and use of Laboratory animals from University of Mysore, Karnataka, India, approved by university committee for animal experiments.

## Conflicts of interest

The authors declare no competing financial interest.

## Supplementary Material

RA-009-C9RA04978A-s001
