# Supplementary material for: Hydrothermal synthesis, characterization and enhanced photocatalytic activity and toxicity studies of a rhombohedral Fe2O3 nanomaterial
Source: RSC Adv. 2019 Aug 13;9(43):25158–69. doi: 10.1039/c9ra04978a (PMC9069891; doi:10.1039/c9ra04978a)
Supplement: RA-009-C9RA04978A-s001 [file RA-009-C9RA04978A-s001.pdf]

Electronic Supplementary Material (ESI) for RSC Advances.
This journal is © The Royal Society of Chemistry 2019

RSC Advances

PAPER

Hydrothermal synthesis, characterization and enhanced photocatalytic activity and toxicity studies of rhombohedral Fe_2O_3 nanomaterial

Abhilash Mavinakere Ramesh, Akshatha Gangadhar, Jagadish Krishnegowda, Mahendra Chikkamadaiah and Srikantaswamy Shivanna

SUPPLEMENTARY INFORMATION

RESEARCH HIGHLIGHTS

1. Facile Hydrothermal synthesis of Rhombohedral Fe_2O_3 for multi-functional application with eco-friendly approaches to compare already reported papers.
2. The synthesized material has high structural uniqueness, high surface area and narrow band gap for the increased photocatalytic performance and recycling performance after photodegradation of Rhodamine-B (RB). The XRD patterns of Rhodamine-B (RB): before and after successful cycles recycling processes, it gives a clear picture on recyclability of the material.
3. First report on, the recycled material was used for toxicity assessments like, histological alterations of the heart tissue of *Albino wistar* rat, *Musmusculus* skin melanoma cells (B16-F10), Human embryonic kidney (HEK)-293, and *Escherichia coli,* (MTCC 7410) bacterial cell wall damage for clear understand of before and after mechanism of material, and this material is safest and the superlative alternative in terms of toxicity in biological application in future
4. Finally, the combination of materials was high photocatalytic stability, non-toxicity and efficient up-conversion properties in the synthesized material paves the way for its applications in photocatalysis, energy conversion and in the biological field as well as environmental degradation and these results are evident gives an encouraging result to compare to already reported materials.

Please do not adjust margins

LIST OF TABLES

Table S1 The elemental composition of Rhombohedral Fe_2O_3 photocatalyst determined from energy dispersive spectroscopy (EDS)

Element	Weight (%)	Atomic (%)	compound
O K	197.03	51.90	270.99
Fe L	293.61	22.15	0.00
C	73.96	25.95	0.00
**Total**		**564.60**	

Table S2 Surface (BET) and pore volume parameters of Rhombohedral Fe_2O_3

Photocatalyst	S_{BET} ($m^2\ g^{-1}$)	Pore volume ($cm^3\ g^{-1}$)
**Fe_2O_3**	5.676	1.304

Table S3 Electro-negativity, band-gap, conduction band (CB) edge and valence band (VB) edge potential of the catalysts on normalized hydrogen scale of Rhombohedral Fe_2O_3

Semiconductor catalyst	x (eV)	E_g (eV)	E_{CB} (eV)	E_{VB} (eV)
**Fe_2O_3**	5.89	1.96	-0.22	2.354

Table S4 The Total organic carbon (TOC) and Chemical oxygen demand (COD) results of Rhodamine - B against Rhombohedral Fe_2O_3

Time (min)	COD (ppm)	TOC (ppm)
**00**	149.12	242.05
**20**	117.39	170.27
**40**	89.74	97.64
**60**	34.97	56.37
**80**	20.41	11.22
**100**	3.54	6.31
**120**	1.39	2.97

This journal is © The Royal Society of Chemistry 2019

Please do not adjust margins

Please do not adjust margins

Table S5 The Anti-bacterial activity of Fe_2O_3 nanoparticle against *E. coli*

**Test Sample**	**Zone of inhibition (in mm)**		
	***E. coli* (0.3 mg)**	***E. coli* (0.6 mg)**	***E. coli* (1.0 mg)**
**Fe_2O_3**	15.81± 0.15	18.35 ± 0.08	20.60± 0.24
**Std. DW**	00.00 ± 0.00	00.00 ± 0.00	00.00 ± 0.00

Note: Values are means of three independent replicates; ± indicate standard error.

	**IC_{50} (µg/ml)**
**Rhombohedral Fe_2O_3**	202.14 ± 0.14
**STD (Cisplatin)**	>500

The data represents in mean ± SD of three experiments

Table S6 Inhibitory concentration values (IC_{50}) of HEK-293 (human embryonic kidney – 293) cells against Rhombohedral Fe_2O_3 nanoparticles.

Table S7 The Photocatalytic degradation intermediates of Rhodamine – B (RB-B) by LC-MS.

Product Code	M/Z	Chemical Formula	Name	Structure
**1 RB-B**	105	$C_7H_5O^+$	benzylidyneoxonium	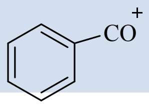
**2 RB-B**	122	$C_7H_6O_2$	benzoic acid	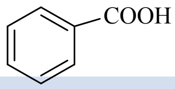
**3 RB-B**	149	$C_9H_{11}NO$	(E)-4-amino-2-(prop-1-en-1-yl)phenol	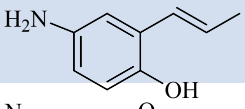
**4 RB-B**	210	$C_{13}H_{10}N_2O$	3-imino-3H-xanthen-6-amine	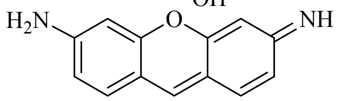
**5 RB-B**	238	$C_{15}H_{14}N_2O$	(Z)-3-(ethylimino)-3H-xanthen-6-amine	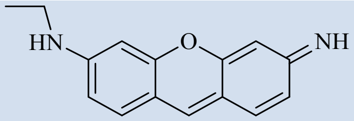
**6 RB-B**	323	$C_{22}H_{29}N_2O^+$	(E)-N,N-diethyl-3-(diethylimmonium)-3H-xanthen-6-amine	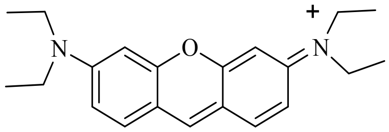

Please do not adjust margins

**7 RB-B**	330	$C_{20}H_{14}N_2O_3$	2-(6-amino-3-imino-3H-xanthen-9-yl)benzoic acid	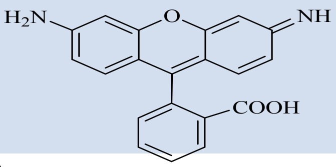
**8 RB-B**	358	$C_{22}H_{18}N_2O_3$	2-(6-(ethylamino)-3-imino-3H-xanthen-9-yl)benzoic acid	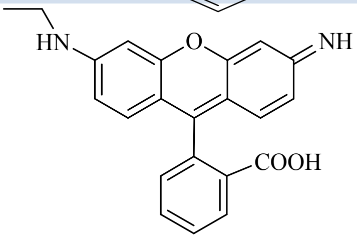
**9 RB-B**	387	$C_{24}H_{22}N_2O_3$	2-(6-(diethylamino)-3-imino-3H-xanthen-9-yl)benzoic acid	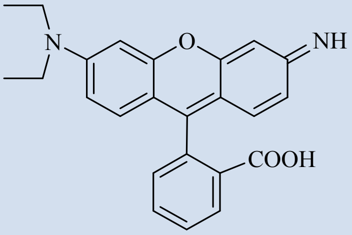

This journal is © The Royal Society of Chemistry 2019
Please do not adjust margins

Please do not adjust margins

LIST OF FIGURES

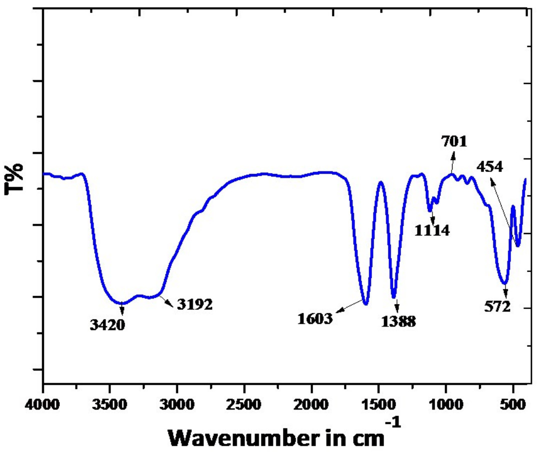

Fig. S1 FT-IR spectrum of the Fe_2O_3 material.

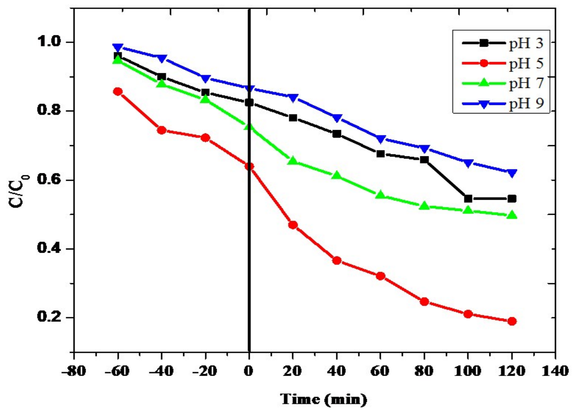

Fig. S2 Role of pH photocatalytic degradation of Rhodamine-B (RB-B) by rhombohedralFe_2O_3.

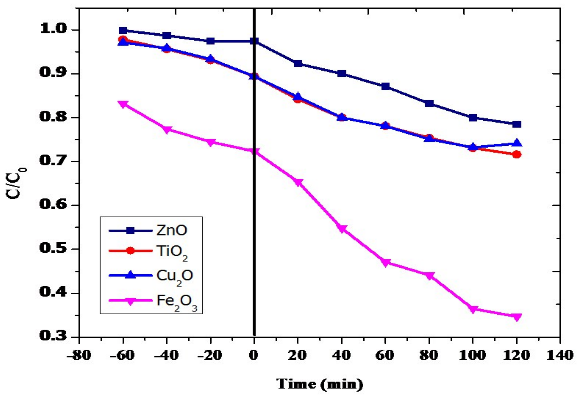

Fig. S3 Photocatalytic degradation of Rhodamine-B (RB-B) using various photocatalysts.

Please do not adjust margins

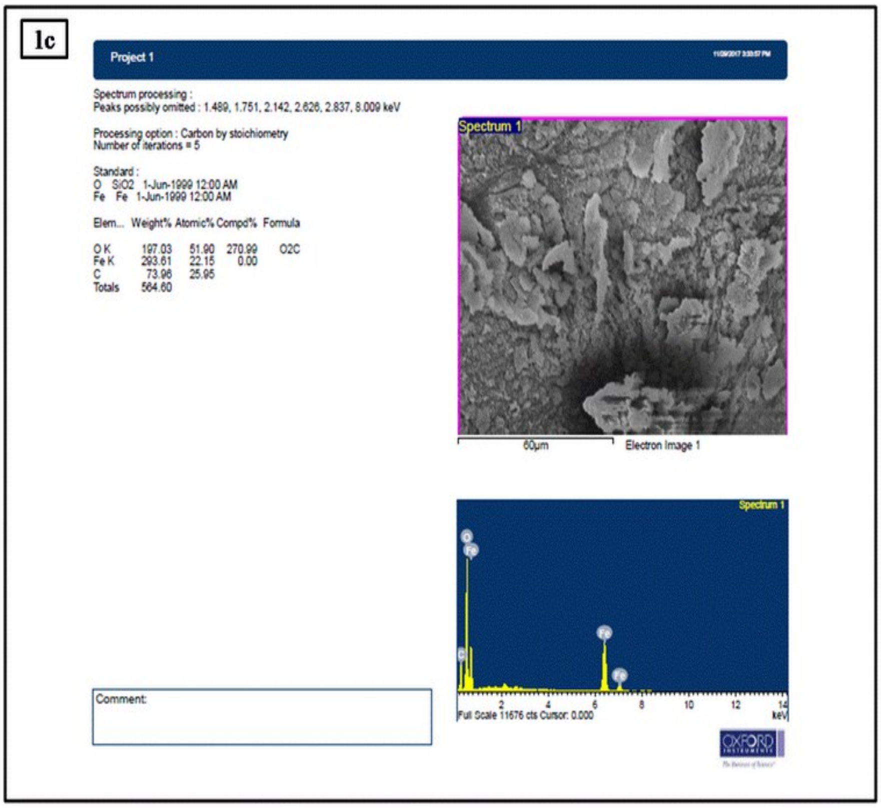

Fig. S4 The elemental composition and atomic percentage of Fe_2O_3in EDS (Sample code: 1c)

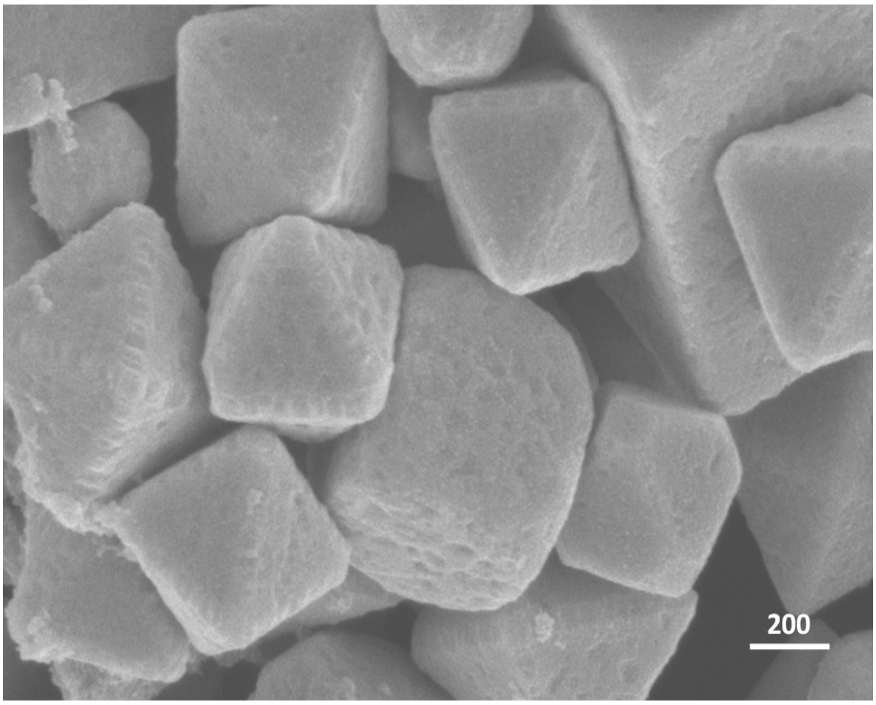

Fig. S5 The FE-SEM image of Rhombohedral Fe_2O_3

This journal is © The Royal Society of Chemistry 2019

Please do not adjust margins

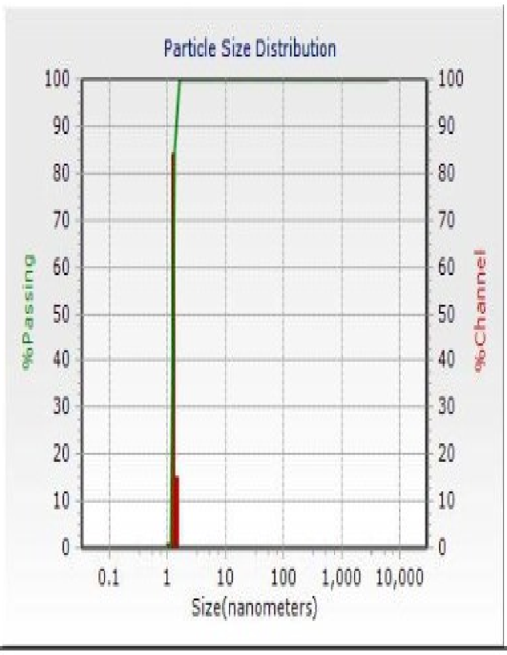

Zeta Potential	
Mobility	1.09u/s/V/cm
Zeta Potential	13.9 mv
Charge	0.00011 fC
Polarity	Positive
Conductivity	388 uS/cm
Field Strength	5.0 kV/m
Sample Information	
Fluid	
Viscosity	0.794
Temperature	30.20 C
Dielectric Const	79
Dispersant	
pH	7
Concentration	
Particle	
Concentration	0

- Particle Size Summary -

Summary	
Data	Value
MV(nm):	1.280
MN(nm):	1.260
MA(nm):	1.270
CS:	4,717
SD:	0.0800
PDI:	11,49,092
Mz:	1.275
si:	0.0996
Ski:	204.9
Kg:	1,036

Percentiles	
%Tile	Size(nm)
10.00	1.160
20.00	1.180
30.00	1.210
40.00	1.230
50.00	1.250
60.00	1.280
70.00	1.300
80.00	1.330
90.00	1.370
95.00	1.420

Peaks Summary		
Dia(nm)	Vol%	Width
1.25	100	0.16

- Measurement Information -

Title	
Particle Size Analysis	
Identifiers	
A-2	
A-2	
Database Record	3
Run Number	1 of 1
Date	23-03-2017
Time	10:53
Acquired Date	23-03-2017
Acquired Time	10:53
Serial Number	W3231
Calculated Data	
Above Residual	0
Below Residual	0
Loading Index	0.001
Conc. Index	6.11
RMS Residual	2.494%
Cell Temp (C)	30.20
Viscosity(cp)	0.7940
Reflected Pwr (uW)	2.89
Recalculation Status	
Live-Meas : : Original :	

Fig. S6 The Rhombohedral Fe_2O_3 Particle size distribution and summery

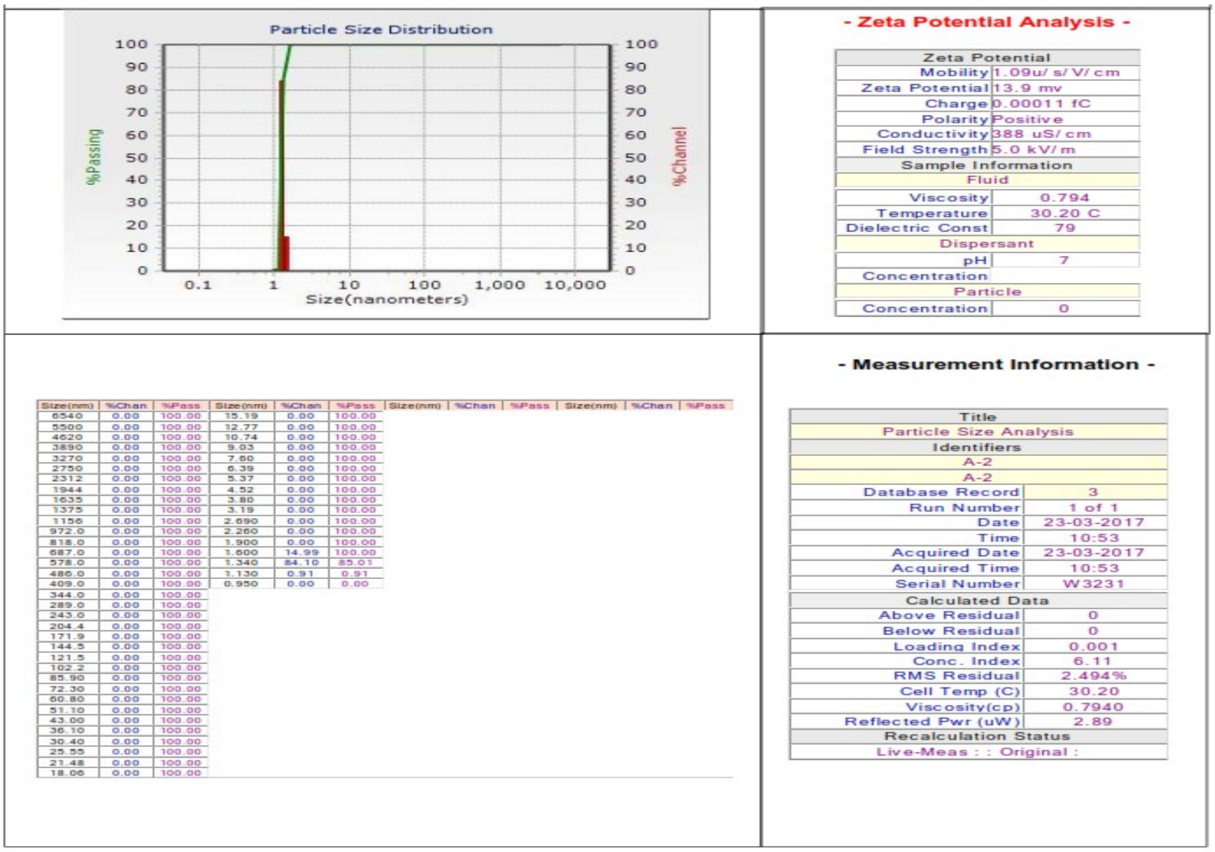

Zeta Potential	
Mobility	1.09u/ s/ V/ cm
Zeta Potential	13.9 mv
Charge	0.00011 fC
Polarity	Positive
Conductivity	388 uS/ cm
Field Strength	5.0 kV/ m
Sample Information	
Fluid	
Viscosity	0.794
Temperature	30.20 C
Dielectric Const	79
Dispersant	
pH	7
Concentration	
Particle	
Concentration	0

Size(nm)	%Chan	%Pass	Size(nm)	%Chan	%Pass	Size(nm)	%Chan	%Pass	Size(nm)	%Chan	%Pass
6540	0.00	100.00	15.19	0.00	100.00						
5500	0.00	100.00	12.77	0.00	100.00						
4620	0.00	100.00	10.74	0.00	100.00						
3890	0.00	100.00	9.03	0.00	100.00						
3270	0.00	100.00	7.60	0.00	100.00						
2750	0.00	100.00	6.39	0.00	100.00						
2312	0.00	100.00	5.37	0.00	100.00						
1944	0.00	100.00	4.52	0.00	100.00						
1635	0.00	100.00	3.80	0.00	100.00						
1375	0.00	100.00	3.19	0.00	100.00						
1156	0.00	100.00	2.690	0.00	100.00						
972.0	0.00	100.00	2.260	0.00	100.00						
818.0	0.00	100.00	1.900	0.00	100.00						
687.0	0.00	100.00	1.600	14.99	100.00						
578.0	0.00	100.00	1.340	84.10	85.01						
486.0	0.00	100.00	1.130	0.91	0.91						
409.0	0.00	100.00	0.950	0.00	0.00						
344.0	0.00	100.00									
289.0	0.00	100.00									
243.0	0.00	100.00									
204.4	0.00	100.00									
171.9	0.00	100.00									
144.5	0.00	100.00									
121.5	0.00	100.00									
102.2	0.00	100.00									
85.90	0.00	100.00									
72.30	0.00	100.00									
60.80	0.00	100.00									
51.10	0.00	100.00									
43.00	0.00	100.00									
36.10	0.00	100.00									
30.40	0.00	100.00									
25.55	0.00	100.00									
21.48	0.00	100.00									
18.06	0.00	100.00									

Title	
Particle Size Analysis	
Identifiers	
A-2	
A-2	
Database Record	3
Run Number	1 of 1
Date	23-03-2017
Time	10:53
Acquired Date	23-03-2017
Acquired Time	10:53
Serial Number	W3231
Calculated Data	
Above Residual	0
Below Residual	0
Loading Index	0.001
Conc. Index	6.11
RMS Residual	2.494%
Cell Temp (C)	30.20
Viscosity(cp)	0.7940
Reflected Pwr (uW)	2.89
Recalculation Status	
Live-Meas : : Original :	

Fig. S7 The Rhombohedral Fe_2O_3 zeta potential analysis results

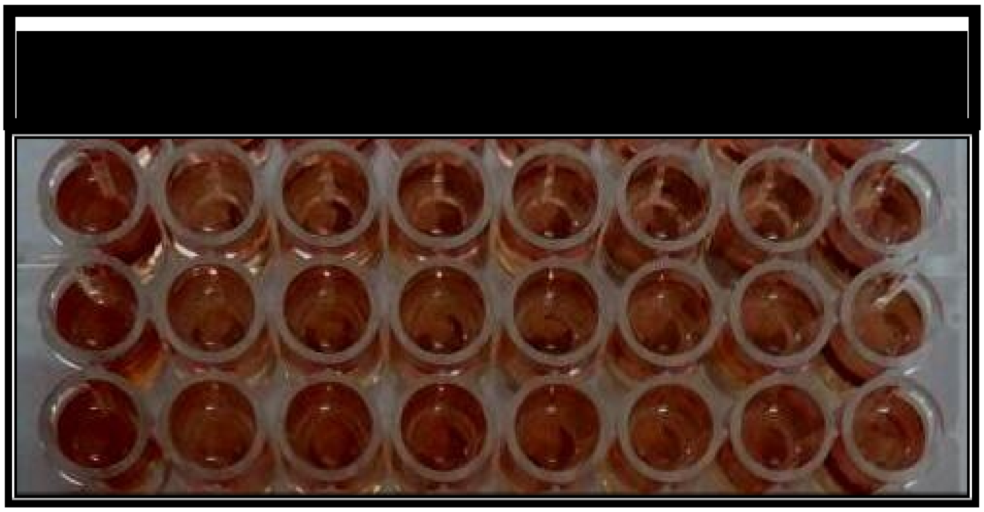

Fig. S8 Cell line activity of Fe_2O_3 by using Human Embryonic Kidney cells – 293 (HEK - 293) at 48 Hours. Cells were grown in 96-well plates in a final volume of 100 µL of culture medium per well. Each well contained 1×10^5 cells/mL (In triplicate to maintain the accuracy as well as to get the better results) and was incubated for 24 h in a 5% CO_2 incubator at 37°C.

 This journal is © The Royal Society of Chemistry 2019

Please do not adjust margins

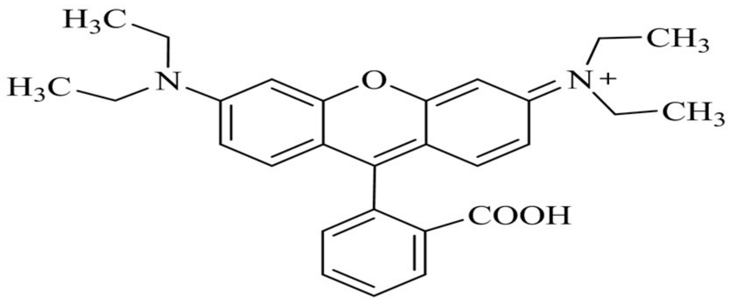

Fig. S9 The Rhombohedral structure

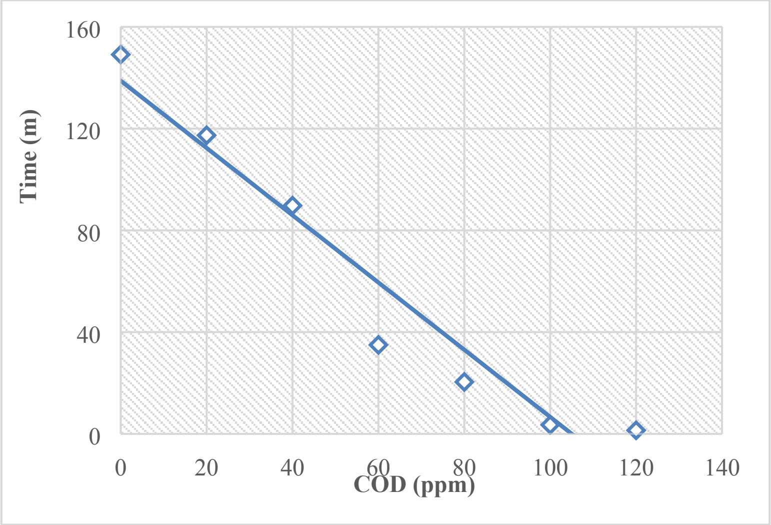

Fig. S10 The negative correlation result of COD vs. time, (Decreased level of COD with increasing time).

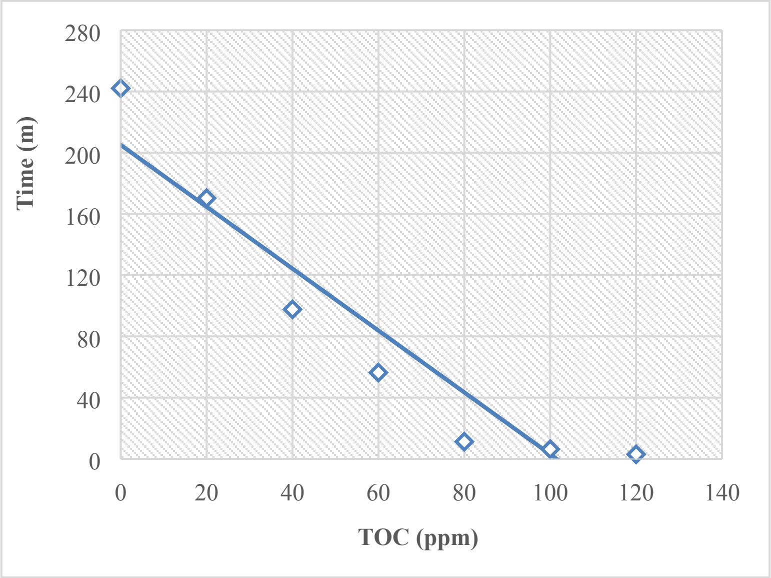

Fig. S11 The negative correlation result of TOC vs. time, (Decreased level of TOC with increasing time).

This journal is © The Royal Society of Chemistry 2019

Please do not adjust margins

Please do not adjust margins

LIST OF INFORMATION

Info. S1 B16-F10 Cell culture and treatment

The (B16-F10) seeded in tissue culture flasks and full-grown in Dulbecco's modified Eagle's medium (DMEM; Thermo Fisher, USA Gibco), balance with 5% fetal bovine serum (FBS, Thermo Fisher; Gibco) and 1% penicillin/streptomycin blend (Santa Cruz Biotechnology, USA). A dose-dependent response curve with different concentrations (0.01, 0.05, 0.1, 0.5, 1.0, and 2.5μg/l) was plotted using propagation and MTT [(2-(3, 5-diphenyltetrazol-2-ium-2-yl)-4,5-dimethyl1-1,3-thiazole bromide)] assay, after 72 hours of exposure. For this, 105 cells were seeded in 24-well plates and cultured with fresh culture medium for 72 hours prior to treatment. Fe_2O_3 be diluted in di-methyl sulfoxide (DMSO; maximum 1% final concentration) and, for this basis, 1% of DMSO was added in the culture medium of the control group. The concentrations of Fe_2O_3 used in the other trails were 5, 50, 100, 200, 300, 400 and 500 μg/l, and they were monitored for duration of 72 hour. All the experiments were performed in triplicate and three independent repetitions.

Info. S2 Normal Cell line toxicity testing using HEK-293

HEK-293 (human embryonic kidney), cell lines were gotten from the American Type Culture Collection (ACC), USA. The growing media: RPMI-1640, Mediated essential medium (MEM), and the phosphate buffer solution (PBS) tablets were obtained from Sigma Chemical Co., St Louis, USA. High quality fetal serum of calf penicillin–streptomycin, trypsin and XTT kit were taken from Sigma labs. Towards perform a cell proliferation assay (XTT) with one 96 wells microplate(1×10^5 cells/mL/well) were incubated for 24 hours in a 5% CO_2 incubator at 37°C. Same methodology involved in HEK-293 cell line IC_{50} assessment, parallelly the test Fe_2O_3 nanomaterial inhibits the 50% of the proliferating the normal cells as per above environmental conditions for 48 hours. The control and effected cell growth were captured using (Olympus, USA).

This journal is © The Royal Society of Chemistry 2019

Please do not adjust margins
